# Milling Stability Prediction Considering Axial Geometric Contact Effects

**DOI:** 10.3390/mi17080977

**Published:** 2026-08-19

**Authors:** Yanlong Zhang, Xiaoru Ren, Junfeng Yang

**Affiliations:** School of Mechatronic Engineering, Lanzhou Jiaotong University, Lanzhou 730070, China; 11240258@stu.lzjtu.edu.cn (X.R.); 15591742090@163.com (J.Y.)

**Keywords:** third-order Lagrange interpolation, Cubic Hermite interpolation, milling, stability lobe diagram, axial contact angle

## Abstract

To overcome the limitations of existing three-degree-of-freedom milling stability models in representing axial cutting conditions, this study develops a stability prediction framework that accounts for both axial segmentation and the axial contact angle. A three-degree-of-freedom dynamic model of the milling system is first formulated by introducing the axial contact angle. The tool axis is then discretized, so that the cutting force coefficients can be evaluated in different axial sections and the non-uniform distribution of cutting forces along the tool can be captured more accurately. After incorporating the regenerative mechanism, the milling dynamics are expressed in the form of a linear time-delay differential equation. To enhance the numerical accuracy of the time-delay system solution, a full-discretization scheme using third-order Lagrange–Hermite interpolation is developed for constructing the state transition matrix. The stability boundary is subsequently determined based on Floquet theory, from which the stability lobe diagram is generated. The proposed model and solution procedure are validated by comparison with existing methods and by time-domain simulation. The results show that, when the spindle speed ranges from 5000 to 10,000 rpm and the axial depth of cut ranges from 0 to 8 mm, the overall variation rate of the predicted stability region is 11.19% after incorporating axial discretization and 59.88% after considering the axial contact angle. The stable and unstable cutting responses obtained from time-domain simulations are consistent with the regions predicted by the stability lobe diagram, which supports the validity of the proposed approach. Further investigation shows that, for the established three-degree-of-freedom milling model and the specified cutting parameters, the axial contact angle exerts a pronounced nonlinear effect on the stability boundary. Specifically, as the axial contact angle ε increases within the range $0^{{}^{\circ}}<\varepsilon \leq 45^{{}^{\circ}}$, the stable region gradually shrinks; when ε increases from 45° to 90°, the stable region expands instead. These observations can provide useful guidance for selecting milling parameters and identifying stable machining conditions.

## 1. Introduction

As advanced manufacturing technologies continue to evolve toward higher precision, greater efficiency, and performance optimization, physics-based modeling, numerical simulation, and experimental validation have been widely applied to investigate complex machining mechanisms and optimize process parameters [1,2,3,4]. In milling, cutting chatter induced by the dynamic interaction between the tool and workpiece is a major factor affecting machining stability. It can deteriorate surface quality and dimensional accuracy, accelerate tool wear, and, in severe cases, damage machine-tool components and limit material removal rate and machining efficiency [5,6,7,8]. Therefore, establishing reliable milling dynamic models and achieving accurate chatter prediction are of great importance.

Regenerative chatter in milling is commonly described by introducing a time-delay term into the cutting-force model to represent the system dynamics, which can be appropriately simplified and expressed as a delay differential equation (DDE) [9]. Based on this dynamic model and appropriate stability criteria, the stability lobe diagram (SLD) can be constructed to determine the stable cutting regions under different combinations of spindle speed and axial depth of cut [10]. Since the SLD provides an intuitive representation of the relationship between process parameters and machining stability, its computational accuracy directly affects the reliability of chatter prediction. Therefore, accurate construction of stability lobe diagrams is a fundamental issue in milling dynamics modeling and stability analysis.

At present, milling chatter stability analysis is mainly conducted using frequency-domain and time-domain methods [11]. Among them, time-domain methods have attracted considerable attention because of their flexibility in handling periodic coefficients, regenerative time delays, and complex dynamic coupling. With the aim of improving stability prediction accuracy, convergence performance, and computational efficiency, existing time-domain approaches can generally be classified into three categories: discrete methods based on high-order interpolation, discrete methods based on numerical integration, and other high-order numerical approximation methods.

The first category mainly improves local approximation accuracy and algorithmic convergence by increasing the interpolation order of the state and time-delay terms. Yan et al. [12] employed third-order Newton interpolation to approximate both the state and delay terms, and achieved third-order convergence through appropriate interpolation-position design. Ma et al. [13] combined spline interpolation with third-order Newton interpolation to approximate the relevant terms in the governing equations and obtained stability lobe diagrams based on Floquet theory. Huang et al. [14] proposed a third-order full-discretization method based on Newton and Hermite interpolation (NI–HI–3rd FDM) for regenerative milling chatter prediction. Xiao et al. [15] used third-order Newton interpolation to treat the delay term and analyzed the convergence performance of the method within the framework of Floquet theory. Zhou et al. [16] reconstructed the state variables using third-order Hermite interpolation, in which derivative information was directly obtained from the original delay differential equations, while an improved numerical integration scheme was adopted for the delay term. Ji et al. [17] further extended the method to multifactor-coupled milling systems and established a full-discretization analysis framework using fourth-order Hermite interpolation and third-order Newton interpolation. High-order interpolation methods can improve the approximation accuracy and convergence performance of state variables and delay terms, but they incur higher computational costs in high-dimensional complex systems.

The second category mainly employs numerical integration strategies to improve the discretization accuracy of the integral terms in the governing equations, while seeking a balance between prediction accuracy and computational efficiency. Yan et al. [18] combined the two-step Simpson integration scheme with the semi-discretization method, constructed the state transition matrix using USBM and UASM, and verified the solution accuracy through convergence analysis. Yan et al. [19] applied a composite high-order full-discretization framework (CHFDM) to single-delay milling systems and further extended it to the computation of stability lobe diagrams for multiple-delay systems. Ji et al. [20] combined Newton interpolation with Simpson integration, representing the delay term and the periodic coefficient matrix by corresponding operators over two adjacent time intervals, and constructed the state transition matrix based on Floquet theory. Yang et al. [21] combined various interpolation polynomials with direct precise integration methods and proposed a full-discretization method based on numerical integration (HFDM). Xia et al. [22] constructed a numerical integration formulation using Lagrange polynomials and Euler’s formula and proposed the numerical integration Euler method (NIEM) to improve the efficiency of milling stability prediction. Xie et al. [23] adopted an Euler-based discretization strategy to uniformly discretize the periodic coefficient matrix, delay term, time-domain term, and differential term. Li et al. [24] transformed the integral equation into a system of algebraic equations using Newton–Cotes quadrature for stability analysis. Zheng et al. [25], on the basis of spatial discretization of the tool and workpiece, established a high-dimensional lumped-parameter dynamic model and employed numerical integration methods for systematic stability analysis and prediction. Numerical integration methods offer good flexibility and prediction accuracy. However, for high-dimensional, multi-delay, and strongly coupled systems, more discretization nodes or higher-order integration schemes are often required, resulting in increased computational cost.

The third category further improves numerical approximation capability by introducing high-order quadrature, exponential fitting, differential quadrature, and related strategies. He et al. [26] employed Clenshaw–Curtis quadrature to discretize the governing equations over the delay interval and determined system stability based on Floquet theory. Wu et al. [27] proposed high-order implicit exponential fitting methods for state approximation, including the third-order IEM and fourth-order LEM, which demonstrated favorable performance in milling dynamics prediction. Mei et al. [28] developed a local differential quadrature method (LDQM) framework for milling stability analysis. These methods offer advantages in approximation accuracy and convergence rate. However, most existing validations are based on conventional or relatively simplified milling dynamics models, and their applicability and stability prediction performance in more complex three-dimensional coupled systems require further investigation. In addition to numerical solution methods, the complexity of the dynamic model itself also directly affects the predicted stability results. Qin et al. [29] established a tool–workpiece–auxiliary support system dynamic model to investigate variations in milling stability under different structural coupling conditions. Ji et al. [17] and Zheng et al. [25] further extended conventional milling stability analysis frameworks from the perspectives of multifactor coupling and high-dimensional dynamic modeling, respectively. These studies indicate that, as practical machining conditions become increasingly complex, merely increasing the order of numerical discretization is insufficient to guarantee reliable stability prediction. Whether the model can adequately represent the actual spatial dynamic characteristics is equally important.

Overall, existing high-order interpolation and numerical integration methods have significantly improved the accuracy and convergence performance of regenerative milling stability analysis. However, under complex machining conditions such as thin-walled components and complex curved surfaces, axial tool vibration and three-dimensional engagement effects become more pronounced. Conventional two-degree-of-freedom models are therefore insufficient to accurately capture the three-directional coupled dynamic characteristics, and the applicability of high-order full-discretization methods to three-degree-of-freedom systems has not yet been systematically investigated. To address the problems of three-dimensional coupled chatter stability prediction and chatter-free parameter selection in complex milling, this study establishes a three-degree-of-freedom dynamic model incorporating three-directional vibration and three-dimensional tool engagement and proposes a third-order Lagrange–Hermite full-discretization method to improve the approximation accuracy of delay terms, numerical convergence performance, and the reliability of stability-boundary prediction. The proposed method provides a theoretical basis for chatter suppression and stable parameter selection in complex machining processes involving thin-walled components and complex curved surfaces.

## 2. Dynamic Model

### Three-Degree-of-Freedom Milling Model

**Figure 1:**
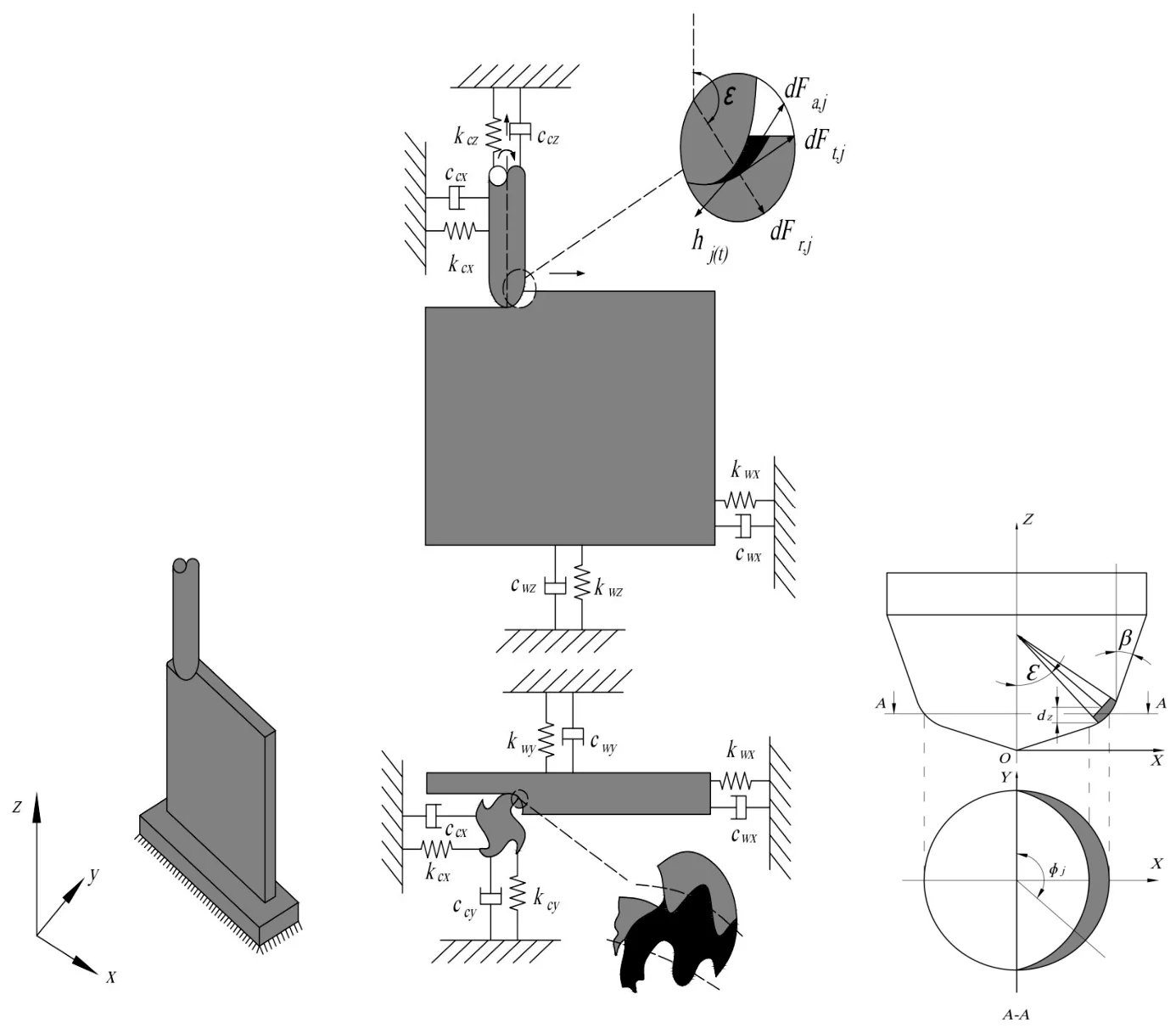
Dynamic model of the three-degree-of-freedom milling system.

The milling dynamic model considering the axial contact angle ε is illustrated in Figure 1. The axial contact angle ε is defined as the angle between the local cutting contact direction and its projection onto the x–y plane. It is assumed that both the cutting tool and the workpiece are flexible bodies. The milling vibration system is defined in three mutually orthogonal directions x, y and z. $k_{cx}$, $k_{cy}$ and $k_{cz}$ denote the stiffness coefficients of the tool in the x, y and z directions, respectively; $k_{wx}$, $k_{wy}$ and $k_{wz}$ denote the stiffness coefficients of the workpiece in the x, y and z directions, respectively; $c_{cx}$, $c_{cy}$, and $c_{cz}$ denote the damping coefficients of the tool in the x, y and z directions, respectively; and $c_{wx}$, $c_{wy}$ and $c_{wz}$ denote the damping coefficients of the workpiece in the x, y and z directions, respectively. $F_{r,j}$, $F_{t,j}$ and $F_{a,j}$ represent the radial, tangential, and axial cutting forces acting on the *j*-th tooth, respectively.

The instantaneous chip thickness considering dynamic effects can be expressed as:

(1) $$h_{j}(t)=g\left(\phi_{j}(t)\right)\left(f_{t}\sin\left(\phi_{j}(t)\right)+{\left[\begin{array}{c} \sin\phi_{j}(t)\cos\varepsilon \\ \cos\phi_{j}(t)\cos\varepsilon \\ -\sin\varepsilon \end{array}\right]}^{T}\left[\begin{array}{c} \left(x_{c}(t)-x_{c}(t-T)\right)-\left(x_{w}(t)-x_{w}(t-T)\right)\\ \left(y_{c}(t)-y_{c}(t-T)\right)-\left(y_{w}(t)-y_{w}(t-T)\right)\\ \left(z_{c}(t)-z_{c}(t-T)\right)-\left(z_{w}(t)-z_{w}(t-T)\right) \end{array}\right]\right)$$

In Equation (1), $f_{t}$ denotes the feed per tooth of the cutter; T represents the time delay; ε is the axial contact angle; and $\phi_{j}(t)$ is the instantaneous engagement angle of the j-th tooth, ${\left[\begin{array}{ccc} x_{c}\left(t\right) & y_{c}\left(t\right) & z_{c}\left(t\right) \end{array}\right]}^{T}$ and ${\left[\begin{array}{ccc} x_{w}\left(t\right) & y_{w}\left(t\right) & z_{w}\left(t\right) \end{array}\right]}^{T}$ denote the displacement vectors of the cutter and the thin-walled workpiece at time t, respectively. which can be expressed as follows:

(2) $$\phi_{j}(t)=\frac{2\pi n}{60}t+\frac{2\pi (j-1)}{N_{t}}$$

In Equation (2), $N_{t}$ denotes the number of teeth, n represents the spindle speed, and j denotes the j-th tooth.

(3) $$g(\phi_{j}(t))=\left\{\begin{array}{cc} 1 & \phi_{\mathrm{st}}\leq \phi_{j}(t)\leq \phi_{\mathrm{ex}}\\ 0 & \mathrm{otherwise} \end{array}\right.$$

In Equation (3), $\phi_{\mathrm{st}}$ and $\phi_{\mathrm{ex}}$ denote the entry angle and exit angle of the cutter tooth, respectively. For up-milling, $\phi_{\mathrm{st}}=\arccos\left(2a_{e}/D-1\right)$, $\phi_{\mathrm{ex}}=\pi$; for down-milling, $\phi_{\mathrm{ex}}=\arccos\left(1-2a_{e}/D\right)$, $\phi_{\mathrm{st}}=0$; $a_{e}/D$ represents the ratio of radial depth of cut to cutter diameter.

The cutting forces acting on the *j*-th tooth, including radial force $F_{r,j}$, tangential force $F_{t,j}$, and axial force $F_{a,j}$, can be expressed as:

(4) $$\left.\begin{array}{l} dF_{r,j}\left[\phi_{j}\left(t\right)\right]=K_{\mathrm{re}}dz+K_{\mathrm{rc}}h_{j}\left(t\right)dz\\ dF_{t,j}\left[\phi_{j}\left(t\right)\right]=K_{\mathrm{te}}dz+K_{\mathrm{tc}}h_{j}\left(t\right)dz\\ dF_{a,j}\left[\phi_{j}\left(t\right)\right]=K_{\mathrm{ae}}dz+K_{\mathrm{ac}}h_{j}\left(t\right)dz \end{array}\right\}$$

In Equation (4), $a_{p}$ denotes the axial depth of cut; $K_{\mathrm{tc}}$, $K_{\mathrm{rc}}$ and $K_{\mathrm{ac}}$ are the cutting force coefficients in the tangential, radial, and axial directions, respectively; $K_{\mathrm{te}}$, $K_{\mathrm{re}}$ and $K_{\mathrm{ae}}$ are the edge force coefficients associated with the tangential, radial, and axial directions, respectively.

The thickness of each axial discretization element is $dz=a_{p}/N_{z}$, where $N_{z}$ the number of axial discretization elements. In the present study, the angle is assumed to remain constant along the axial direction.

The radial, tangential, and axial cutting forces $F_{r,j}$, $F_{t,j}$ and $F_{a,j}$ can then be resolved into the x, y and z directions as follows:

(5) $$\left[\begin{array}{c} dF_{x,j}\\ dF_{y,j}\\ dF_{z,j} \end{array}\right]=\left[\begin{array}{ccc} -\sin\varepsilon \cos\phi_{j}(t) & -\cos\phi_{j}(t) & -\cos\varepsilon \sin\phi_{j}(t)\\ -\sin\varepsilon \sin\phi_{j}(t) & -\sin\phi_{j}(t) & -\cos\varepsilon \cos\phi_{j}(t)\\ \cos\varepsilon & 0 & -\sin\varepsilon \end{array}\right]\left[\begin{array}{c} dF_{t,j}\\ dF_{r,j}\\ dF_{a,j} \end{array}\right]$$

When the cutter has multiple teeth, the cutting forces acting on all teeth are summed to obtain the resultant cutting force components of the cutter in the x, y and z directions:

(6) $$F(t)=\left[\begin{array}{c} F_{x}\\ F_{y}\\ F_{z} \end{array}\right]=\sum_{j=0}^{N_{t}-1}\sum_{l=0}^{N_{z}-1}g\left(\phi_{j}(t)\right)\left[\begin{array}{ccc} -\sin\varepsilon \cos\phi_{j}(t) & -\cos\phi_{j}(t) & -\cos\varepsilon \sin\phi_{j}(t)\\ -\sin\varepsilon \cos\phi_{j}(t) & -\sin\phi_{j}(t) & -\cos\varepsilon \cos\phi_{j}(t)\\ \cos\varepsilon & 0 & -\sin\varepsilon \end{array}\right]dz\left\{\left[\begin{array}{c} K_{\mathrm{tc}}\\ K_{\mathrm{rc}}\\ K_{\mathrm{ac}} \end{array}\right]h_{j}(t)+\left[\begin{array}{c} K_{\mathrm{te}}\\ K_{\mathrm{re}}\\ K_{\mathrm{ae}} \end{array}\right]\right\}$$

In general, the three-degree-of-freedom milling dynamic model considering the regenerative effect can be formulated as a time-delay differential equation:

(7) $$M\left[\begin{array}{c} \ddot{x}(t)\\ \ddot{y}(t)\\ \ddot{z}(t) \end{array}\right]+C\left[\begin{array}{c} \dot{x}(t)\\ \dot{y}(t)\\ \dot{z}(t) \end{array}\right]+K\left[\begin{array}{c} x(t)\\ y(t)\\ z(t) \end{array}\right]=\left[\begin{array}{ccc} -a_{p}h_{xx}(t) & -a_{p}h_{xy}(t) & -a_{p}h_{xz}(t)\\ -a_{p}h_{yx}(t) & -a_{p}h_{yy}(t) & -a_{p}h_{yz}(t)\\ -a_{p}h_{zx}(t) & -a_{p}h_{zy}(t) & -a_{p}h_{zz}(t) \end{array}\right]\left[\begin{array}{c} x(t)\\ y(t)\\ z(t) \end{array}\right]+\left[\begin{array}{ccc} a_{p}h_{xx}(t) & a_{p}h_{xy}(t) & a_{p}h_{xz}(t)\\ a_{p}h_{yx}(t) & a_{p}h_{yy}(t) & a_{p}h_{yz}(t)\\ a_{p}h_{zx}(t) & a_{p}h_{zy}(t) & a_{p}h_{zz}(t) \end{array}\right]\left[\begin{array}{c} x(t-T)\\ y(t-T)\\ z(t-T) \end{array}\right].$$

In the equation, M, C and K denote the modal mass, damping, and stiffness matrices of the system, respectively.

$$h_{xx}(t)=\sum_{j=0}^{N_{t}-1}\sum_{l=0}^{N_{z}-1}\frac{dz}{\sin\varepsilon }g(\phi_{j}(t))\sin\phi_{j}(t)\cos\varepsilon \left[K_{\mathrm{tc}}\sin\varepsilon \cos\phi_{j}(t)+K_{\mathrm{rc}}\cos\phi_{j}(t)+K_{\mathrm{ac}}\cos\varepsilon \sin\phi_{j}(t)\right]$$

$$h_{xy}(t)=\sum_{j=0}^{N_{t}-1}\sum_{l=0}^{N_{z}-1}\frac{dz}{\sin\varepsilon }g(\phi_{j}(t))\cos\phi_{j}(t)\cos\varepsilon \left[K_{\mathrm{tc}}\sin\varepsilon \cos\phi_{j}(t)+K_{\mathrm{rc}}\cos\phi_{j}(t)+K_{\mathrm{ac}}\cos\varepsilon \sin\phi_{j}(t)\right]$$

$$h_{xz}(t)=-\sum_{j=0}^{N_{t}-1}\sum_{l=0}^{N_{z}-1}\frac{dz}{\sin\varepsilon }g(\phi_{j}(t))\sin\varepsilon \left[K_{\mathrm{tc}}\sin\varepsilon \cos\phi_{j}(t)+K_{\mathrm{rc}}\cos\phi_{j}(t)+K_{\mathrm{ac}}\cos\varepsilon \sin\phi_{j}(t)\right]$$

$$h_{yx}(t)=\sum_{j=0}^{N_{t}-1}\sum_{l=0}^{N_{z}-1}\frac{dz}{\sin\varepsilon }g(\phi_{j}(t))\sin\phi_{j}(t)\cos\varepsilon \left[K_{\mathrm{tc}}\sin\varepsilon \cos\phi_{j}(t)-K_{\mathrm{rc}}\sin\phi_{j}(t)+K_{\mathrm{ac}}\cos\varepsilon \cos\phi_{j}(t)\right]$$

$$h_{yy}(t)=\sum_{j=0}^{N_{t}-1}\sum_{l=0}^{N_{z}-1}\frac{dz}{\sin\varepsilon }g(\phi_{j}(t))\cos\phi_{j}(t)\cos\varepsilon \left[K_{\mathrm{tc}}\sin\varepsilon \cos\phi_{j}(t)-K_{\mathrm{rc}}\sin\phi_{j}(t)+K_{\mathrm{ac}}\cos\varepsilon \cos\phi_{j}(t)\right]$$

$$h_{yz}(t)=\sum_{j=0}^{N_{t}-1}\sum_{l=0}^{N_{z}-1}\frac{dz}{\sin\varepsilon }g(\phi_{j}(t))\sin\varepsilon \left[K_{\mathrm{tc}}\sin\varepsilon \cos\phi_{j}(t)-K_{\mathrm{rc}}\sin\phi_{j}(t)+K_{\mathrm{ac}}\cos\varepsilon \cos\phi_{j}(t)\right]$$

$$h_{zx}(t)=\sum_{j=0}^{N_{t}-1}\sum_{l=0}^{N_{z}-1}\frac{dz}{\sin\varepsilon }g(\phi_{j}(t))\sin\phi_{j}(t)\cos\varepsilon \left[-K_{\mathrm{tc}}\cos\varepsilon +K_{\mathrm{ac}}\sin\varepsilon \right]$$

$$h_{zy}(t)=\sum_{j=0}^{N_{t}-1}\sum_{l=0}^{N_{z}-1}\frac{dz}{\sin\varepsilon }g(\phi_{j}(t))\cos\phi_{j}(t)\cos\varepsilon \left[-K_{\mathrm{tc}}\cos\varepsilon +K_{\mathrm{ac}}\sin\varepsilon \right]$$

(8) $$h_{zz}(t)=-\sum_{j=0}^{N_{t}-1}\sum_{l=0}^{N_{z}-1}\frac{dz}{\sin\varepsilon }g(\phi_{j}(t))\sin\varepsilon \left[-K_{\mathrm{tc}}\cos\varepsilon +K_{\mathrm{ac}}\sin\varepsilon \right]$$

In Equation (8), $j=0,1,2\ldots \ldots N_{t}-1$ is the tooth index, $l=0,1,2\ldots \ldots N_{z}-1$ is the axial-segment index, $N_{t}$ is the number of teeth, $N_{z}$ is the number of axial segments, and $dz=a_{p}/N_{z}$ is the axial width of each segment.

Let $p(t)=M\overset{\cdot }{q\left(t\right)}+Cq\left(t\right)/2$ and $s(t)={\left[\begin{array}{cc} q{\left(t\right)}^{T} & p{\left(t\right)}^{T} \end{array}\right]}^{T}$, the state-space representation of the three-degree-of-freedom milling model in Equation (7) can be written as:

(9) $$\dot{s}(t)=A_{0}s\left(t\right)+A\left(t\right)s\left(t\right)+B\left(t\right)s(t\text{-}T)$$

where

$$A_{0}=\left[\begin{array}{cc} -M^{-1}C/2 & M^{-1}\\ CM^{-1}C/4-K & -CM^{-1}/2 \end{array}\right]$$

$$A\left(t\right)=\left(\begin{array}{cccccc} 0 & 0 & 0 & 0 & 0 & 0\\ 0 & 0 & 0 & 0 & 0 & 0\\ 0 & 0 & 0 & 0 & 0 & 0\\ -a_{p}h_{xx}(t) & -a_{p}h_{xy}(t) & -a_{p}h_{xz}(t) & 0 & 0 & 0\\ -a_{p}h_{yx}(t) & -a_{p}h_{yy}(t) & -a_{p}h_{yz}(t) & 0 & 0 & 0\\ -a_{p}h_{zx}(t) & -a_{p}h_{zy}(t) & -a_{p}h_{zz}(t) & 0 & 0 & 0 \end{array}\right)$$

$$B\left(t\right)=\left(\begin{array}{cccccc} 0 & 0 & 0 & 0 & 0 & 0\\ 0 & 0 & 0 & 0 & 0 & 0\\ 0 & 0 & 0 & 0 & 0 & 0\\ a_{p}h_{xx}(t) & a_{p}h_{xy}(t) & a_{p}h_{xz}(t) & 0 & 0 & 0\\ a_{p}h_{yx}(t) & a_{p}h_{yy}(t) & a_{p}h_{yz}(t) & 0 & 0 & 0\\ a_{p}h_{zx}(t) & a_{p}h_{zy}(t) & a_{p}h_{zz}(t) & 0 & 0 & 0 \end{array}\right)$$

The third-order Lagrange–Hermite interpolation-based full-discretization method is employed for milling stability analysis. For $k\tau \leq t\leq \left(k+1\right)\tau$, $\left(k=0,\cdots ,m\right)$, the response of Equation (9) can be expressed as:

(10) $$s(t)=e^{A_{0}(t-k\tau )}s(k\tau )+\int_{k\tau }^{t}\left\{e^{A_{0}(t-\xi )}\left[A(\xi )s(\xi )+B(\xi )s(\xi -T)\right]\right\}d\xi$$

(11) $$s_{k+1}=e^{A_{0}\tau }s(k\tau )+\int_{0}^{\tau }\left\{e^{A_{0}\xi }\left[A(k\tau +\tau -\xi )s(k\tau +\tau -\xi )+B(k\tau +\tau -\xi )s(k\tau +\tau -\xi -T)\right]\right\}d\xi$$

where the state term is approximated using third-order Lagrange interpolation as follows:

(12) $$s(k\tau +\tau -\xi )=\frac{\xi^{3}-3\xi^{2}\tau +2\xi \tau^{2}}{6\tau^{3}}s_{k-2}+\frac{4\xi^{2}\tau -\xi^{3}-3\xi \tau^{2}}{2\tau^{3}}s_{k-1}+\frac{6\tau^{2}\xi -5\xi^{2}\tau +\xi^{3}}{2\tau^{3}}s_{k}+\frac{6\tau^{3}-\xi^{3}-11\tau^{2}\xi +6\tau \xi^{2}}{6\tau^{3}}s_{k+1}$$

The third-order Hermite interpolation is expressed as:

$$s(t)=N_{1}(\theta )s_{i}+N_{2}(\theta )s_{\left(i+1\right)}+N_{3}(\theta )h{\dot{s}}_{i}+N_{4}(\theta )h{\dot{s}}_{\left(i+1\right)}$$

where $\theta =(t-t_{i})/h$.

For the endpoint derivatives, the three-point backward difference scheme is adopted:

$${\dot{s}}_{i}\approx (3s_{i}-4s_{\left(i-1\right)}+s_{\left(i-2\right)})/(2h)$$

By combining third-order Hermite interpolation with the three-point backward difference approximation of the endpoint derivatives, the delayed state s(kτ+τ−ξ−T) is expressed as follows:

(13) $$s(k\tau +\tau -\xi -T)=\left(1-\frac{3\xi }{2\tau }+\frac{\xi^{3}}{2\tau^{3}}\right)s_{k-m+1}+\left(\frac{2\xi }{\tau }+\frac{\xi^{2}}{2\tau^{2}}-\frac{3\xi^{3}}{2\tau^{3}}\right)s_{k-m}+\left(\frac{3\xi^{3}}{2\tau^{3}}-\frac{\xi }{2\tau }-\frac{\xi^{2}}{\tau^{2}}\right)s_{k-m-1}+\left(\frac{\xi^{2}}{2\tau^{2}}-\frac{\xi^{3}}{2\tau^{3}}\right)s_{k-m-2}$$

The one-order linear interpolation of A(kτ+τ−ξ) is:

(14) $$A(k\tau +\tau -\xi )\approx A_{k+1}+\left(A_{k}-A_{k+1}\right)\frac{\xi }{\tau }$$

The one-order linear interpolation of B(kτ+τ−ξ) is:

(15) $$B(k\tau +\tau -\xi )\approx B_{k+1}+\left(B_{k}-B_{k+1}\right)\frac{\xi }{\tau }$$

Substituting the interpolated results into the time-delay differential equation, if the matrix ${\left(I-Q_{i,1}\right)}^{-1}$ is nonsingular, it can be written as:

(16) $$\begin{array}{l} s_{k+1}={\left(I-Q_{k,1}\right)}^{-1}Q_{k,-2}s_{k-2}+{\left(I-Q_{k,1}\right)}^{-1}Q_{k,-1}s_{k-1}+{\left(I-Q_{k,1}\right)}^{-1}\left(Q_{k,0}+H_{0}\right)s_{k}+{\left(I-Q_{k,1}\right)}^{-1}Q_{k,m-1}s_{k-m+1}\\ +{\left(I-Q_{k,1}\right)}^{-1}Q_{k,m}s_{k-m}+{\left(I-Q_{k,1}\right)}^{-1}Q_{k,m+1}s_{k-m-1}+{\left(I-Q_{k,1}\right)}^{-1}Q_{k,m+2}s_{k-m-2} \end{array}$$

where:

$$Q_{k,-2}=\left(\frac{4H_{4}}{6\tau^{3}}-\frac{5H_{3}}{6\tau^{2}}+\frac{H_{2}}{3\tau }-\frac{H_{5}}{6\tau^{4}}\right)A_{k+1}+\left(\frac{H_{5}}{6\tau^{4}}-\frac{H_{4}}{2\tau^{3}}+\frac{H_{3}}{3\tau^{2}}\right)A_{k}$$

$$Q_{k,-1}=\left(\frac{7H_{3}}{2\tau^{2}}-\frac{5H_{4}}{2\tau^{3}}-\frac{3H_{2}}{2\tau }+\frac{H_{5}}{2\tau^{4}}\right)A_{k+1}+\left(\frac{2H_{4}}{\tau^{3}}-\frac{H_{5}}{2\tau^{4}}-\frac{3H_{3}}{2\tau^{2}}\right)A_{k}$$

$$Q_{k,0}=\left(\frac{3H_{2}}{\tau }-\frac{11H_{3}}{2\tau^{2}}+\frac{3H_{4}}{\tau^{3}}-\frac{H_{5}}{2\tau^{4}}\right)A_{k+1}+\left(\frac{3H_{3}}{\tau^{2}}-\frac{5H_{4}}{2\tau^{3}}+\frac{H_{5}}{2\tau^{4}}\right)A_{k}$$

$$Q_{k,1}=\left(H_{1}-\frac{7H_{4}}{6\tau^{3}}-\frac{17H_{2}}{6\tau }+\frac{17H_{3}}{6\tau^{2}}+\frac{H_{5}}{6\tau^{4}}\right)A_{k+1}+\left(\frac{H_{2}}{\tau }-\frac{H_{5}}{6\tau^{4}}-\frac{11H_{3}}{6\tau^{2}}+\frac{H_{4}}{\tau^{3}}\right)A_{k}$$

$$Q_{k,m-1}=\left(H_{1}-\frac{5H_{2}}{2\tau }+\frac{H_{4}}{2\tau^{3}}-\frac{H_{5}}{2\tau^{4}}+\frac{3H_{3}}{2\tau^{2}}\right)B_{k+1}+\left(\frac{H_{2}}{\tau }-\frac{3H_{3}}{2\tau^{2}}+\frac{H_{5}}{2\tau^{4}}\right)B_{k}$$

$$Q_{k,m}=\left(\frac{2H_{2}}{\tau }-\frac{3H_{3}}{2\tau^{2}}-\frac{2H_{4}}{\tau^{3}}+\frac{3H_{5}}{2\tau^{4}}\right)B_{k+1}+\left(\frac{2H_{3}}{\tau^{2}}+\frac{H_{4}}{2\tau^{3}}-\frac{3H_{5}}{2\tau^{4}}\right)B_{k}$$

$$Q_{k,m+1}=\left(\frac{5H_{4}}{2\tau^{3}}-\frac{H_{3}}{2\tau^{2}}-\frac{H_{2}}{2\tau }-\frac{3H_{5}}{2\tau^{4}}\right)B_{k+1}+\left(\frac{3H_{5}}{2\tau^{4}}-\frac{H_{4}}{\tau^{3}}-\frac{H_{3}}{2\tau^{2}}\right)B_{k}$$

(17) $$Q_{k,m+2}=\left(\frac{H_{3}}{2\tau^{2}}-\frac{H_{4}}{\tau^{3}}+\frac{H_{5}}{2\tau^{4}}\right)B_{k+1}+\left(\frac{H_{4}}{2\tau^{3}}-\frac{H_{5}}{2\tau^{4}}\right)B_{k}$$

In Equation (17),

$$H_{0}=e^{A_{0}\tau },\;H_{1}=\int_{0}^{\tau }e^{A_{0}\xi }d\xi ,\;H_{2}=\int_{0}^{\tau }e^{A_{0}\xi }\xi d\xi ,$$

$$H_{3}=\int_{0}^{\tau }e^{A_{0}\xi }\xi^{2}d\xi ,\;H_{4}=\int_{0}^{\tau }e^{A_{0}\xi }\xi^{3}d\xi ,\;H_{5}=\int_{0}^{\tau }e^{A_{0}\xi }\xi^{4}d\xi$$

Therefore, the discrete mapping can be defined as:

(18) $$y_{k+1}=E_{k}y_{k}$$

where the $\left(3m+6\right)$-dimensional vector $y_{k}$ can be expressed as:

(19) $$y_{k}=\mathrm{col}\left(s_{k}s_{k-1}\ldots s_{k-m}s_{k-m-1}s_{k-m-2}\right)$$

$E_{k}$ is defined as:

(20) $$E_{k}=\left[\begin{array}{ccccccc} d_{k} & d_{k\text{-}1} & d_{k\text{-}2} & \cdots & d_{k\text{-}m} & d_{k\text{-}m\text{-}1} & d_{k\text{-}m\text{-}2}\\ I & & & & & & \\ & I & & & & & \\ & & I & & & & \\ & & & \ldots & & & \\ & & & & I & & \\ & & & & & I & \end{array}\right]$$

where:

$$d_{k}={\left(I-Q_{k,1}\right)}^{-1}\left(Q_{k,0}+H_{0}\right)$$

$$d_{k\text{-}1}={\left(I-Q_{k,1}\right)}^{-1}Q_{k,-1}$$

$$d_{k-2}={\left(I-Q_{k,1}\right)}^{-1}Q_{k,-2}$$

…

Using the discrete mapping matrix $E_{k}\left(k=0,1,\ldots m-1\right)$, the transfer matrix Φ over one full period can be constructed as:

(21) $$y_{m}=\Phi y_{0}$$

where Φ is defined as:

(22) $$\Phi =E_{m\text{-}1}E_{m-2}\ldots E_{1}E_{0}$$

Finally, according to Floquet theory, the milling system is considered stable if all eigenvalues of the transfer matrix Φ lie within the unit circle. Otherwise, chatter occurs.

(23) $$\max\left(\left|\lambda \left(\Phi \right)\right|\right)\left\{\begin{array}{cc} >1, & \mathrm{Chatter}\\ =1, & \mathrm{Critical}\;\mathrm{stability}\\ <1, & Stable \end{array}\right.$$

## 3. Numerical Simulation Results and Analysis

### 3.1. Convergence Analysis

For a given discretization number, the dominant Floquet multiplier μ is defined as the eigenvalue of the state-transition matrix having the maximum modulus. Therefore, its modulus is equal to the spectral radius of Φ:

(24) $$\left|\mu \right|=\rho \left(\Phi \right)=\underset{i}{\max}\left|\mu_{i}\right|$$

In Equation (24), $\mu_{i}$ denotes the ith Floquet multiplier of Φ, while Φ represents the state transition matrix.

**Figure 2:**
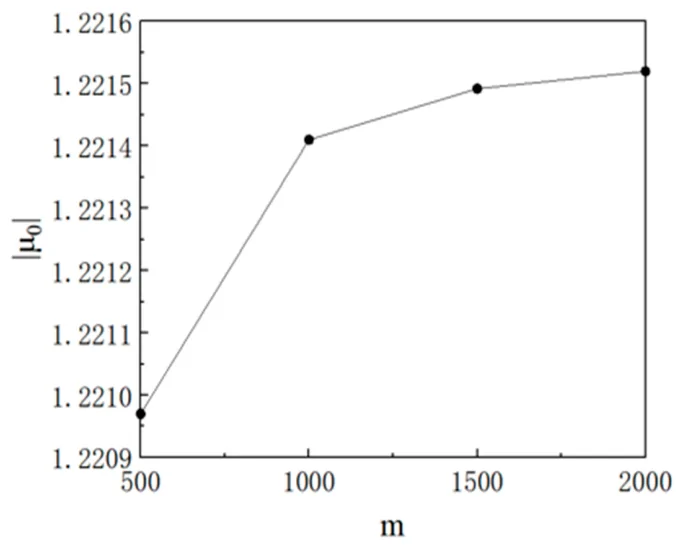
Convergence of the dominant Floquet multiplier modulus calculated by the first-order FDM for different discretization numbers.

When the number of discretization intervals m is sufficiently large, the stability boundary predicted by the first-order full-discretization method (1st-FDM) gradually approaches the converged solution [30]. To obtain a reliable reference solution, mesh-refinement calculations were performed using the 1st-FDM with m=500, 1000, 1500, and 2000, as shown in Figure 2.

Based on the data in Figure 2,

$$\Delta_{2000}=\left|\left|\mu \left(2000\right)\right|-\left|\mu \left(1500\right)\right|\right|=3.0\times 10^{-5}$$

$$\delta_{1500,2000}=\frac{\left|\left|\mu \left(2000\right)\right|-\left|\mu \left(1500\right)\right|\right|}{\left|\mu \left(2000\right)\right|}=2.45596\times 10^{-5}$$

For m=1500,2000, the relative difference between the two results is only $2.45596\times 10^{-5}$ indicating that further refinement of the discretization has a negligible effect on the computed result. Therefore, the result obtained with m=2000 is adopted as the high-resolution numerical reference for the subsequent error analysis,

$$\left|\mu_{0}\right|=\left|\mu_{\mathrm{1st-FDM}}\left(m=2000\right)\right|$$

The corresponding local discretization error is defined as:

(25) $$E_{\mathrm{abs}}(m)=\left|\left|\mu |-|\mu_{0}\right|\right|$$

The relative difference between m = 1500 and m = 2000 is used only to verify the convergence of the reference solution. All local discretization errors reported in this study are absolute errors.

The cutting tool is made of YG6X cemented carbide. The simulation parameters adopted in the present numerical study are consistent with those reported in Ref. [30], namely, $m_{tx}=m_{ty}=m_{tz}=m_{t}=0.03993\mathrm{kg}$, $\zeta_{x}=\zeta_{y}=\zeta_{z}=\zeta =0.011$, $f_{nx}=f_{ny}=f_{nz}=f_{n}=922\mathrm{Hz}$, $k_{x}=m_{tx}f_{nx}f_{nx}$, $k_{y}=m_{ty}f_{ny}f_{ny}$, $k_{z}=m_{tz}f_{nz}f_{nz}$, the other parameter values are provided in Table 1 and Table 2.

**Figure 3:**
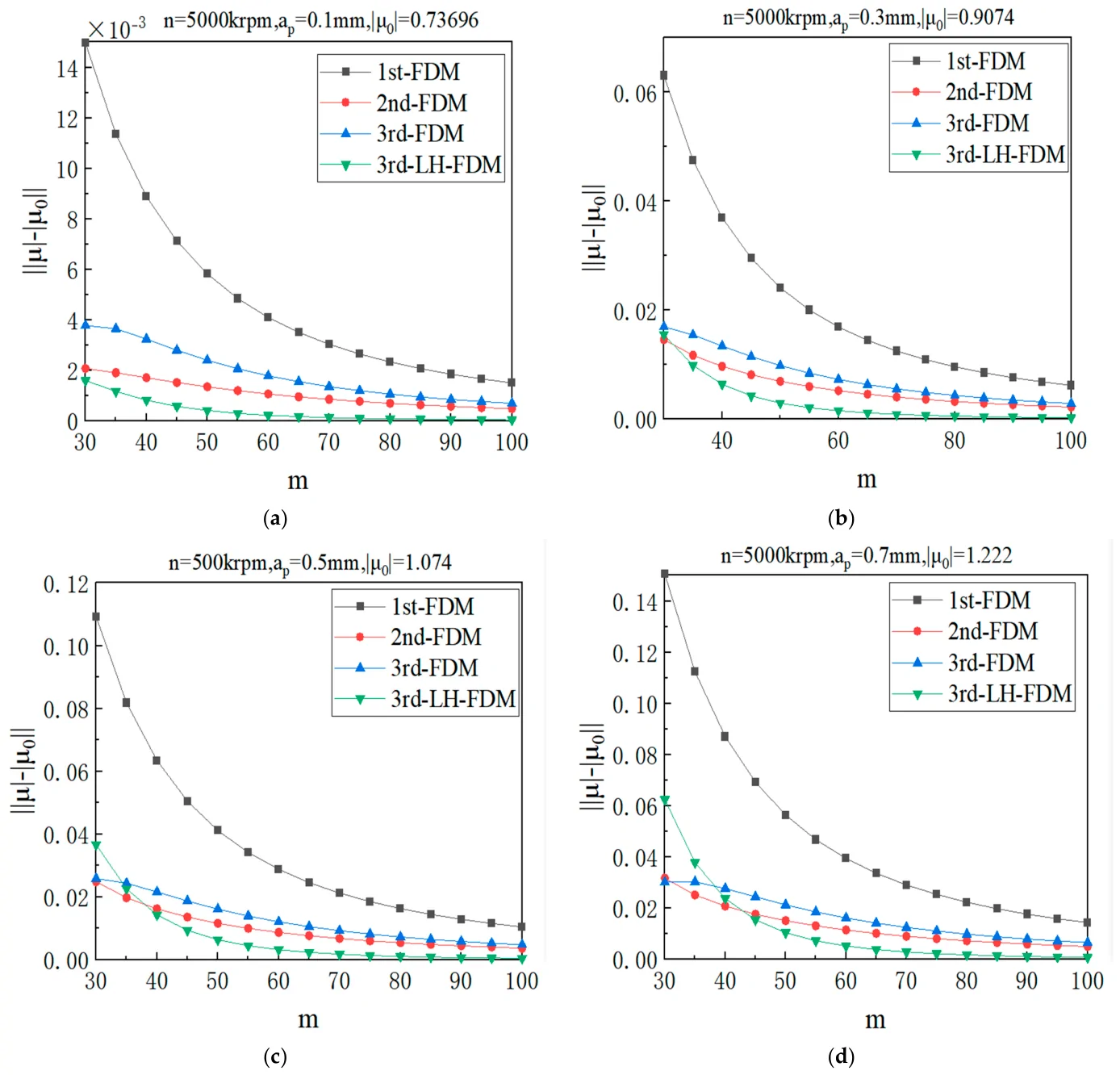
Comparison of convergence rates among 1st-FDM, 2nd-FDM, 3rd-FDM and the 3rd-LH-FDM. (**a**) $a_{p}$ = 0.1 mm. (**b**) $a_{p}$ = 0.3 mm. (**c**) $a_{p}$ = 0.5 mm. (**d**) $a_{p}$ = 0.7 mm.

At four representative axial depths of cut, the local discretization error curves obtained using the first-order full-discretization method (1st-FDM), second-order full-discretization method (2nd-FDM), third-order full-discretization method (3rd-FDM), and the proposed method (3rd-LH-FDM) are compared in Figure 3. As the discretization is progressively refined, i.e., as the number of discretization intervals m increases, the errors of all four methods decrease gradually and approach a negligible level. For a given value of m, the proposed 3rd-LH-FDM consistently yields a smaller local discretization error than the three conventional FDM-based methods, indicating its superior local approximation accuracy.

As a representative example, for a spindle speed of 5000 r/min, an axial depth of cut of 0.3 mm, and m=50, the local discretization errors obtained using the 1st-FDM, 2nd-FDM, 3rd-FDM, and 3rd-LH-FDM are 0.024, 0.007, 0.010, and 0.003, respectively. Compared with the 1st-FDM, 2nd-FDM, and 3rd-FDM, the proposed 3rd-LH-FDM reduces the local discretization error by approximately 87.5%, 57%, and 70%, respectively.

**Figure 4:**
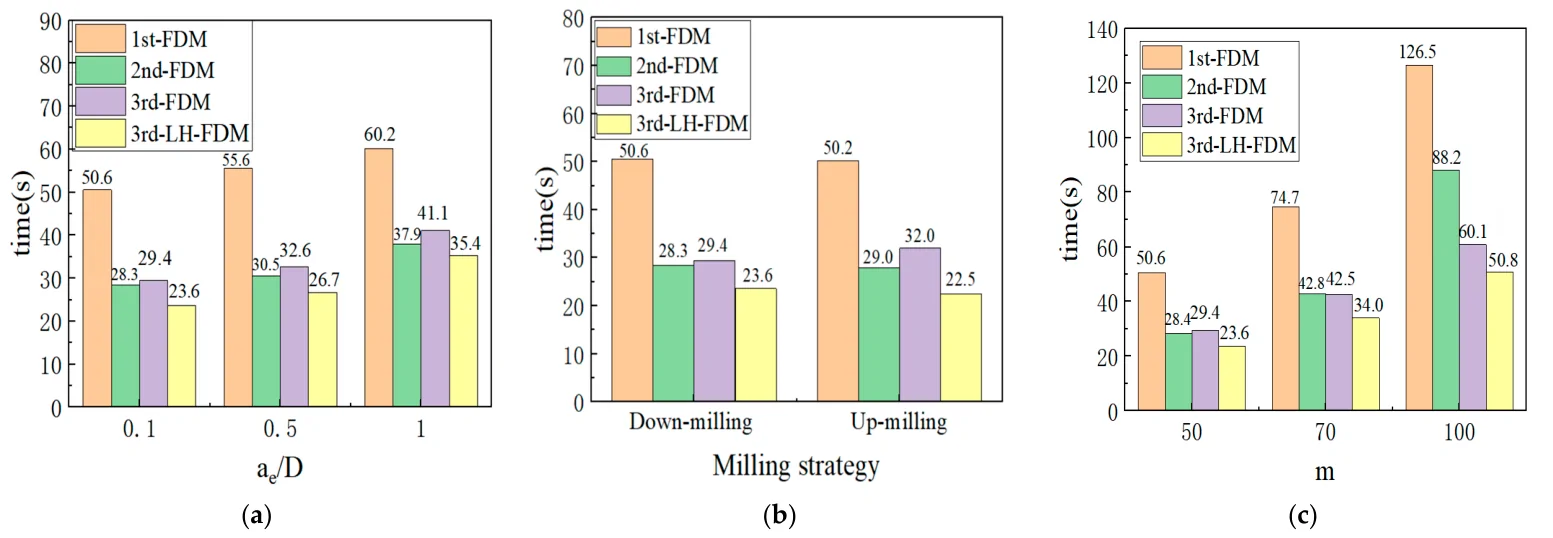
Comparison of the computational time consumed by the 1st-FDM, the 2nd-FDM, the 3rd-FDM, and the 3rd-LH-FDM. (**a**) Different $a_{e}/D$. (**b**) Different milling strategies. (**c**) Different m.

Moreover, for a prescribed error tolerance, the proposed method can achieve the required accuracy with fewer discretization intervals. As shown in Figure 4, when $a_{e}/D=0.1$, the computational time of the 3rd-LH-FDM is reduced by 53.4%, 16.6%, and 19.7% compared with the 1st-FDM, 2nd-FDM, and 3rd-FDM, respectively. These results demonstrate that the proposed method provides a clear computational-efficiency advantage over the three conventional FDM schemes.

### 3.2. Influence of Axial Discretization and Axial Contact Angle

In the three-degree-of-freedom milling model, ε denotes the axial contact angle, and dz/sinε represents the actual cutting length of the cutting edge. The axial micro-element dz is the axial projection of the actual cutting-edge micro-element dl, satisfying dz=dl⋅sinε, thus, dl=dz/sinε. The instantaneous axial depth of cut $a_{p}$ is uniformly divided into $N_{z}=15$ axial elements along the tool axis, and the thickness of each element is $dz=a_{p}/N_{z}=a_{p}/15$. For each axial element, the instantaneous angular position, cutting state, and directional coefficients of the cutter teeth are determined using Equations (2), (3), and (8), respectively. The contributions from all engaged teeth and axial elements are then summed directly. This summation is equivalent to an equal-width rectangular quadrature over the axial depth of cut. The resulting time-varying directional coefficient matrix is used in the subsequent stability analysis.

**Figure 5:**
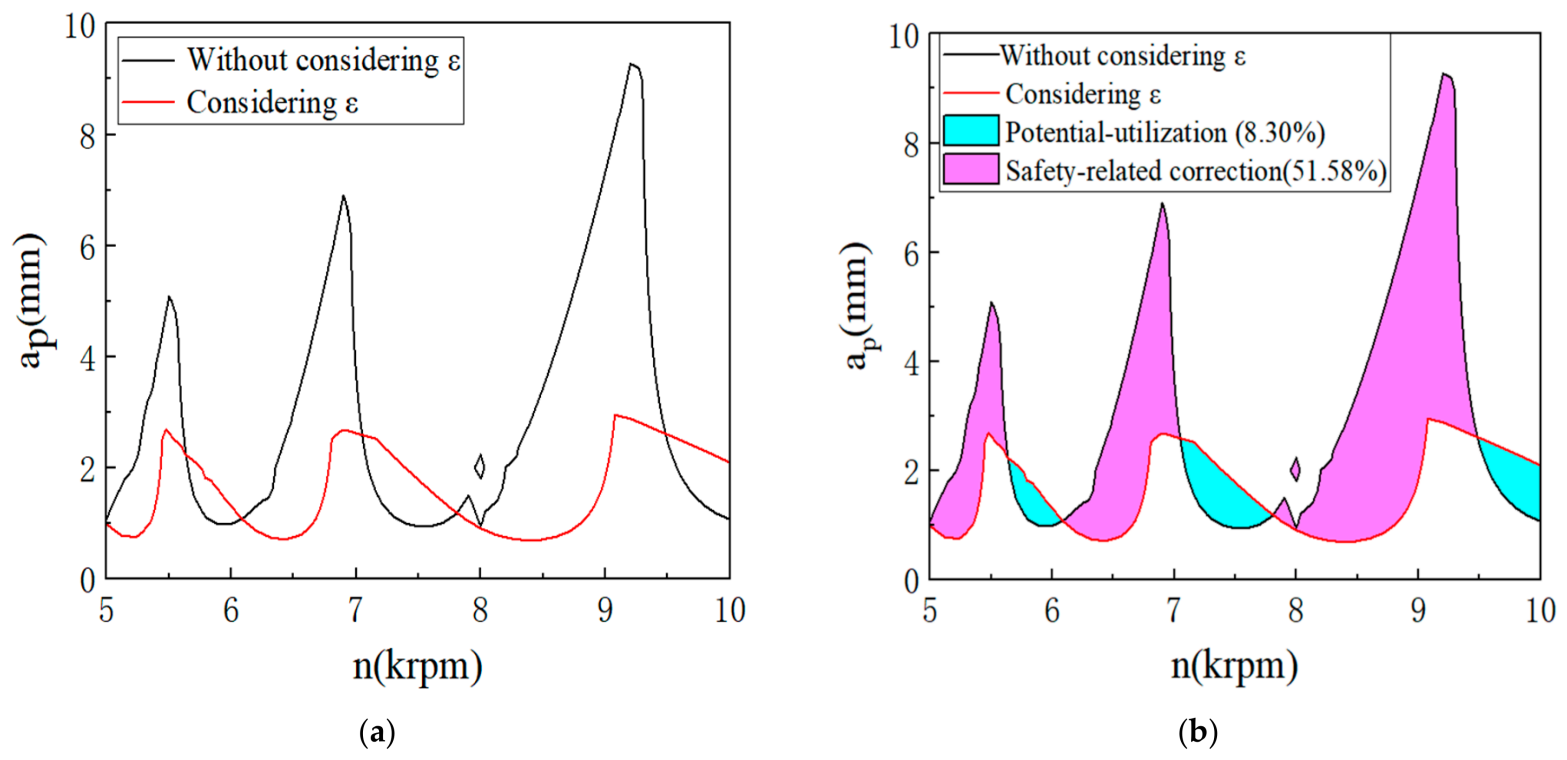
Comparison of stability lobe diagrams with and without axial contact angle ε. (**a**) SLD about ε. (**b**) Region partitioning.

**Figure 6:**
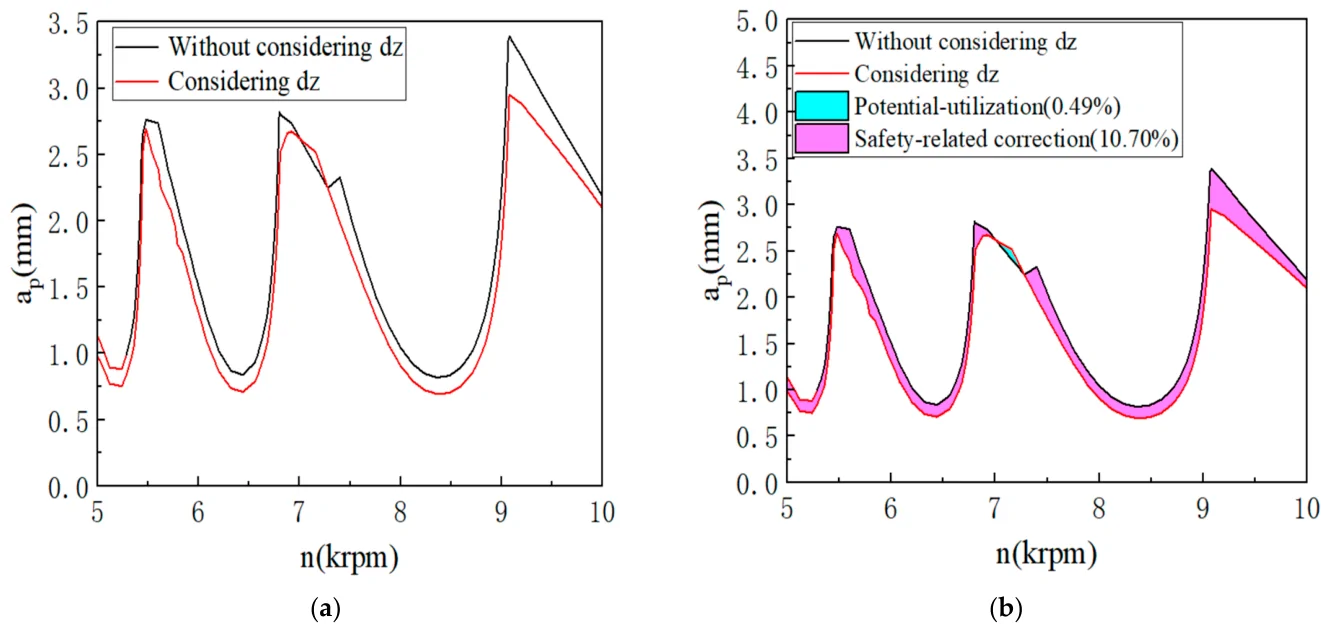
Comparison of stability lobe diagrams with and without axial discretization. (**a**) SLD about dz. (**b**) Region partitioning.

When axial discretization and the axial contact angle are neglected, the critical axial depth of cut in the stability lobe diagram is generally overestimated, whereas considering both factors yields a stability region closer to the actual condition. As shown in Figure 5 and Figure 6, under down-milling conditions with a discretization number of m = 40 and a radial immersion ratio of $a_{e}/D=0.1$, the predicted stability regions are compared for cases with and without consideration of the axial contact angle and axial discretization, respectively. To quantitatively characterize the differences in the predicted stability regions under different modeling conditions, the safety-related correction region and the potential-utilization region are defined.

The safety-related correction region corresponds to the magenta regions in Figure 5 and Figure 6. These regions are predicted to be stable when the axial contact angle or axial discretization is neglected, but are identified as unstable when the corresponding factor is taken into account. The potential-utilization region is shown in blue in Figure 5 and Figure 6. It represents the region predicted to be unstable when the axial contact angle or axial discretization is neglected, but stable when the corresponding factor is included.

To quantitatively evaluate these regions, a two-dimensional rectangular numerical integration method is used over the spindle-speed range of 5000–10,000 rpm and the axial-depth range of 0–8 mm. The grid areas satisfying the corresponding stability criteria are summed to obtain the safety-related correction region and the potential-utilization region. As the reference, the two regions are normalized and defined as follows:

$$P_{\mathrm{safety}}=\frac{S_{\mathrm{safety}}}{S_{0}}\times 100\%$$

(26) $$P_{\mathrm{potential}}=\frac{S_{\mathrm{potential}}}{S_{0}}\times 100\%$$

where $S_{0}$ denotes the stable region predicted by the simplified model, $S_{\mathrm{safety}}$ is the area classified as stable by the simplified model but unstable by the improved model, and $S_{\mathrm{potential}}$ is the area classified as unstable by the simplified model but stable by the improved model. The normalized areas are shown in Figure 5 and Figure 6.

To quantitatively characterize the variation in the predicted stability-region area caused by different modeling factors, the Stable Area Ratio (SAR) is introduced as a normalized evaluation indicator. SAR is essentially a dimensionless ratio between the stability-region area obtained after incorporating a specific factor and that of the baseline model. The degree to which SAR deviates from 1 reflects the relative change in the total stability-region area after incorporating the corresponding factor, while whether SAR is greater than or less than 1 indicates the direction of this change.

The corresponding definitions are given as follows:

(27) $$\mathrm{SAR1}=\frac{S_{dz}}{S_{0}}$$

(28) $$\mathrm{SAR2}=\frac{S_{\varepsilon }}{S_{0}}$$

**Figure 7:**
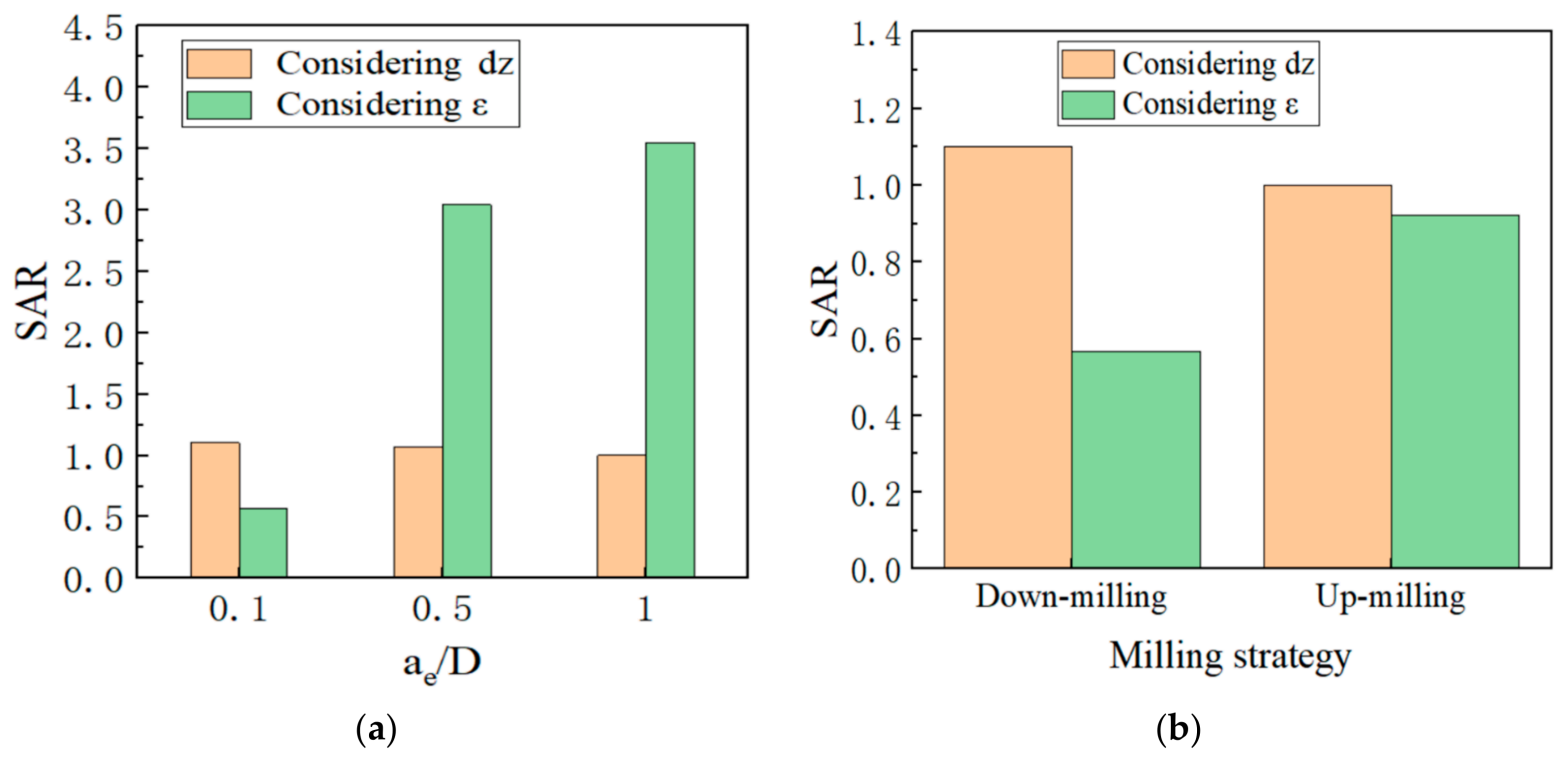
SAR under different milling conditions. (**a**) SAR under different a_e_/D values. (**b**) SAR under different milling strategies.

In Equations (27) and (28), the stability-region ratio obtained by considering axial discretization is denoted by SAR1 (orange box), whereas that obtained by considering the axial contact angle is denoted by SAR2 (green box). $S_{0}$ denotes the stability-region area predicted by the simplified model, while $S_{dz}$ and $S_{\varepsilon }$ represent the stability-region areas obtained by considering axial discretization and the axial contact angle, respectively. An SAR value greater than 1 indicates an increase in the predicted stability-region area relative to that of the simplified model, whereas a value less than 1 indicates a decrease. An SAR value equal to 1 indicates no change in the total stability-region area. The SAR values under different radial immersion ratios and milling strategies are shown in Figure 7.

Based on the above definitions, the stability region in Figure 5a was evaluated using two-dimensional rectangular numerical integration, and the results are shown in Figure 5b. Within the parameter ranges of spindle speed n = 5000–10,000 rpm and axial depth of cut a_p_ = 0–8 mm, after considering the axial contact angle, the safety-related correction region accounts for 51.58% of the stable region predicted by the simplified model, while the potential-utilization region accounts for 8.30%. The overall variation rate of the predicted stability region is 59.88%. The results indicate that, within the above parameter ranges, neglecting the axial contact angle mainly results in an enlargement of the predicted stability region.

For the axial discretization case, as shown in Figure 6, within the parameter ranges of spindle speed n = 5000–10,000 rpm and axial depth of cut a_p_ = 0–8 mm, the safety-related correction region and the potential-utilization region account for 10.70% and 0.49% of the stable region predicted by the simplified model, respectively. The overall variation rate of the predicted stability region is 11.19%. This indicates that axial discretization mainly affects the correction of the overestimated stable region, while its effect on the additional stable region is limited.

As shown in Figure 7, axial discretization has a relatively small effect on the area of the stability region, with the corresponding SAR1 values remaining close to unity under all milling conditions. In contrast, the axial contact angle ε has a more pronounced influence on the area of the stability region, and this influence is closely related to the radial immersion ratio $a_{e}/D$ and the milling strategy. As $a_{e}/D$ increases, SAR2 increases significantly, indicating that neglecting the axial contact angle ε leads to a marked underestimation of the stability-region area at medium and large radial immersion ratios. Under small radial immersion ratios and down-milling conditions, however, SAR2 is smaller than unity, suggesting that neglecting ε may lead to an overestimation of the stability region. Therefore, both the axial contact angle and axial discretization should be considered in milling stability prediction.

### 3.3. Simulation Analysis

**Figure 8:**
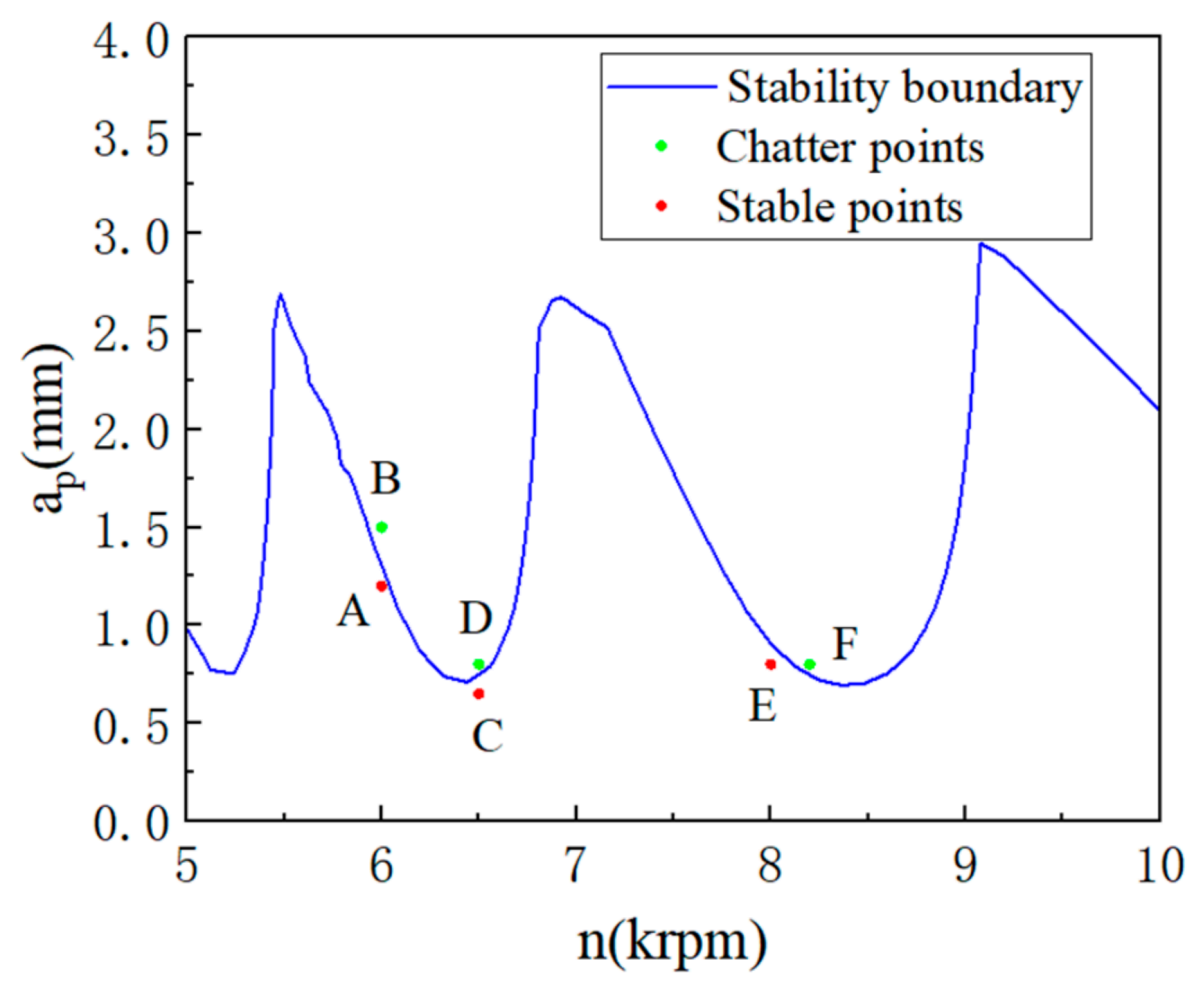
Stability lobe diagram for $a_{e}/D=0.1$, m = 40, and $\varepsilon =75^{\circ }$. A: (6000 rpm, 1.2 mm), B: (6000 rpm, 1.5 mm), C: (6500 rpm, 0.65 mm), D: (6500 rpm, 0.8 mm), E: (8000 rpm, 0.8 mm), and F: (8200 rpm, 0.8 mm).

Figure 8 presents the stability lobe diagram of the milling process.

**Figure 9:**
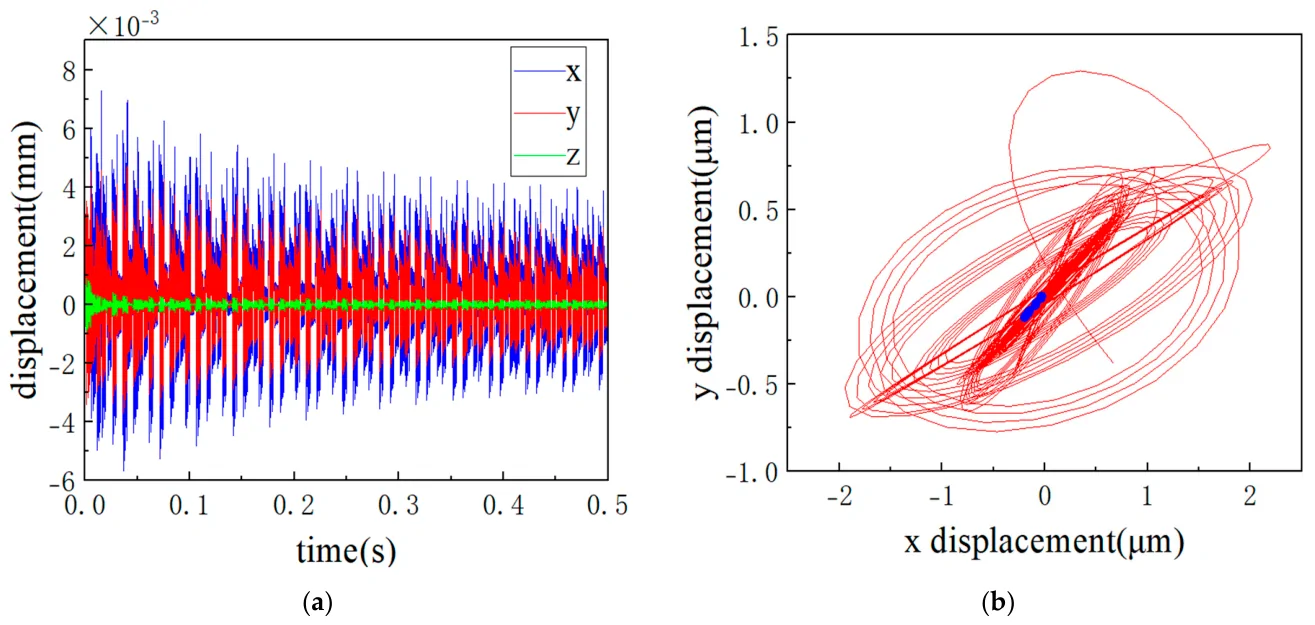
Simulation results at point A. (**a**) Three-directional displacement plot at Point A. (**b**) Phase trajectory (red) with Poincaré points (blue) at Point A.

**Figure 10:**
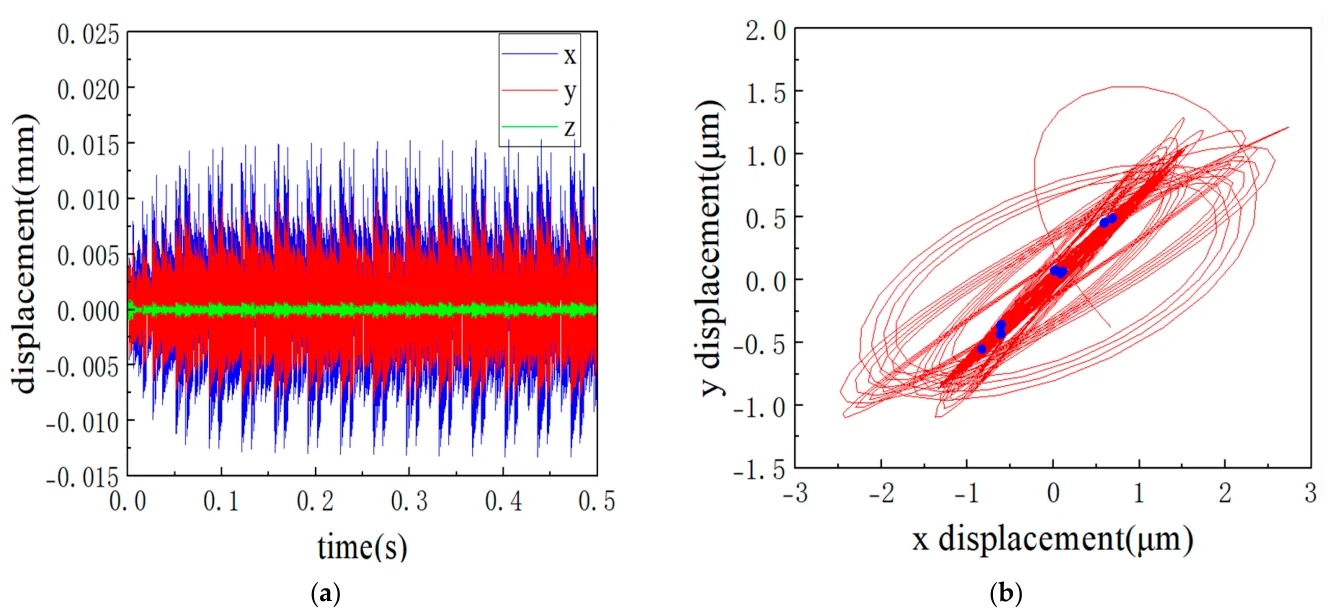
Simulation results at point B. (**a**) Three-directional displacement plot at Point B. (**b**) Phase trajectory (red) with Poincaré points (blue) at Point B.

**Figure 11:**
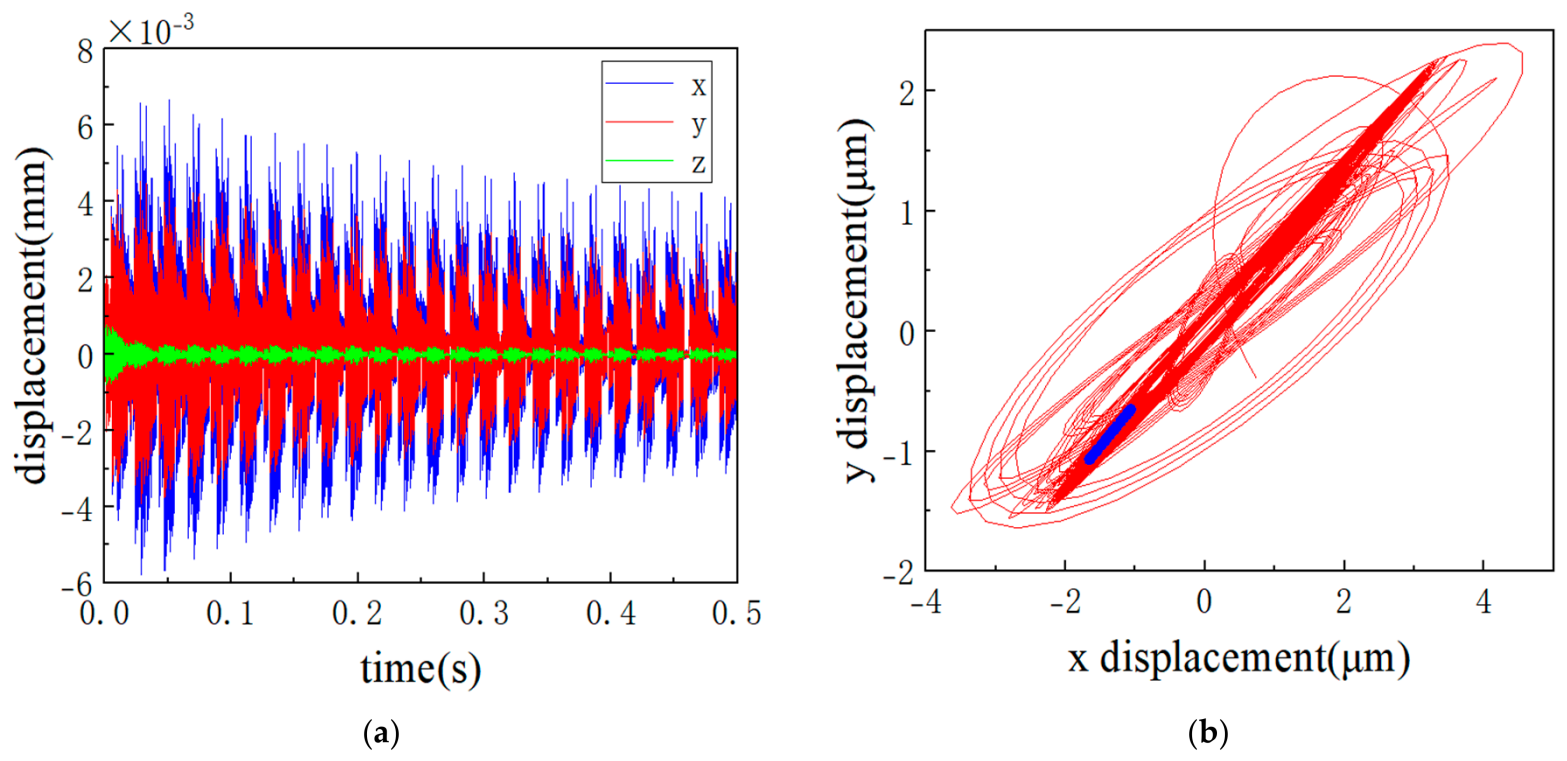
Simulation results at point C. (**a**) Three-directional displacement plot at Point C. (**b**) Phase trajectory (red) with Poincaré points (blue) at Point C.

**Figure 12:**
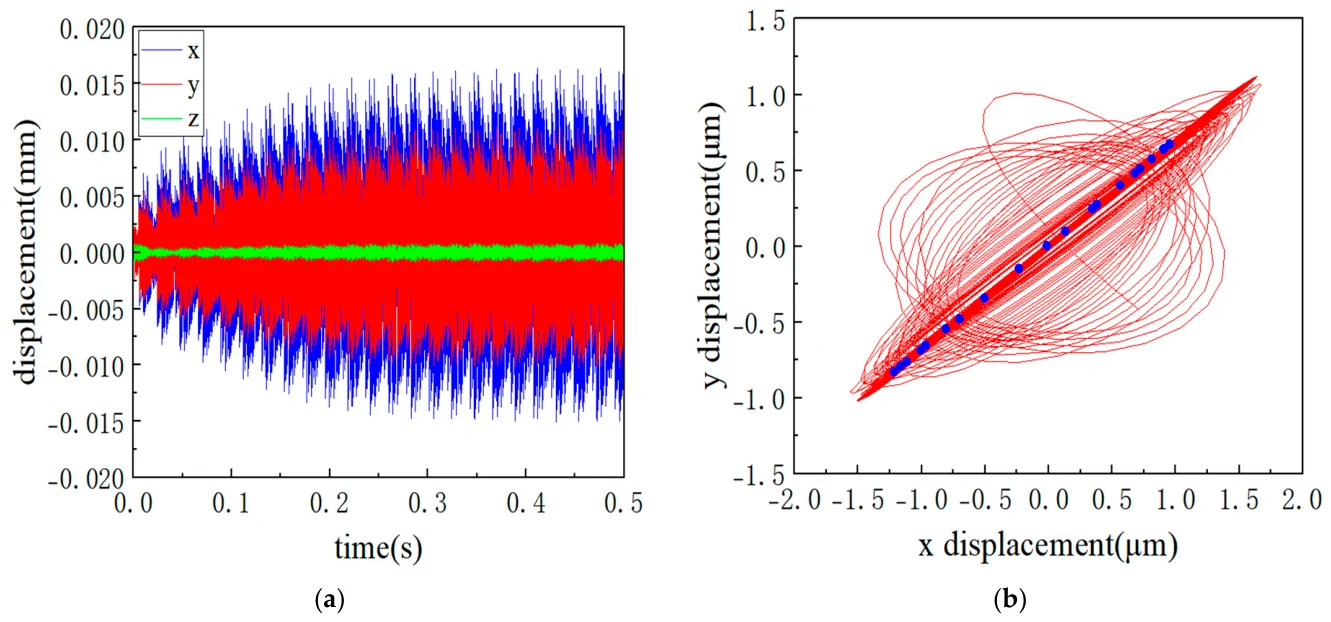
Simulation results at point D. (**a**) Three-directional displacement plot at Point D. (**b**) Phase trajectory (red) with Poincaré points (blue) at Point D.

**Figure 13:**
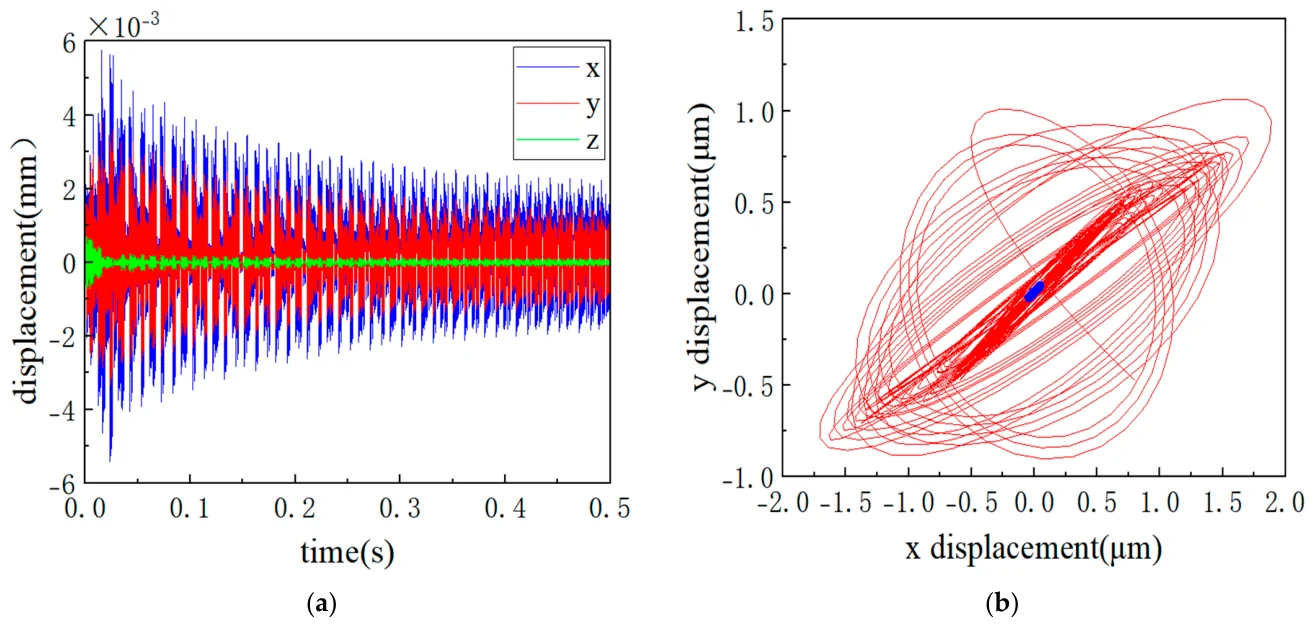
Simulation results at point E. (**a**) Three-directional displacement plot at Point E. (**b**) Phase trajectory (red) with Poincaré points (blue) at Point E.

**Figure 14:**
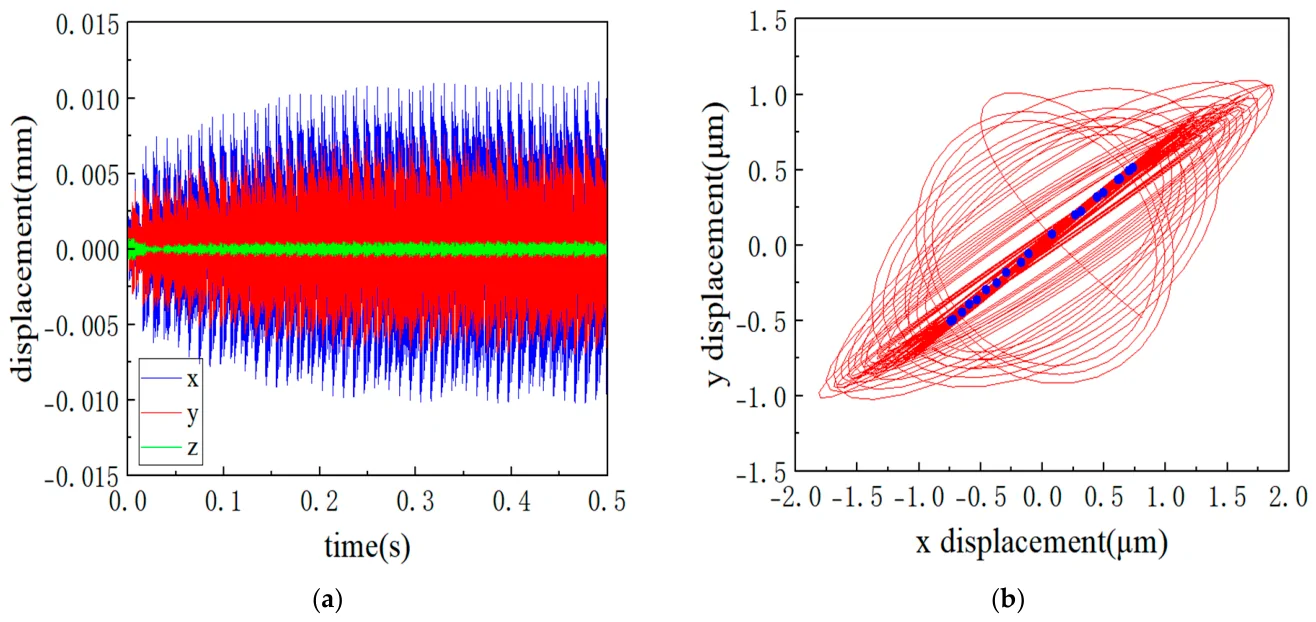
Simulation results at point F. (**a**) Three-directional displacement plot at Point F. (**b**) Phase trajectory (red) with Poincaré points (blue) at Point F.

To validate the accuracy of the milling model, six points are selected from Figure 8. Vibration displacement analysis and phase-plane analysis are then performed to determine the milling stability, as shown in Figure 9, Figure 10, Figure 11, Figure 12, Figure 13 and Figure 14.

A fixed-step classical fourth-order Runge–Kutta (RK4) method is used for the time-domain integration of the three-degree-of-freedom milling delay differential equations. The regenerative cutting force is determined from the displacement difference between the current state and the delayed state one tooth-passing period earlier. For delayed states at intermediate RK4 stages that do not coincide with the discrete time nodes, linear interpolation between adjacent known historical states is used to obtain the required delayed states.

The tooth-passing period is determined from the spindle speed and the number of cutter teeth as

(29) $$T=60/\left(nN_{t}\right)$$

where n is the spindle speed and $N_{t}$ is the number of cutter teeth. Each tooth-passing period is divided into 100 time steps, giving a time step size of

(30) $$\Delta t=\frac{T}{100}$$

The total simulation time is set to 0.5 s. For t≤0, a constant history function is prescribed as the initial history of the delay system, and the initial velocities in the x, y, and z directions are all set to zero.

System stability is jointly determined from the three-direction displacement responses and the phase-plane trajectory with Poincaré sampling points. The system is considered stable when the vibration amplitudes in the x,y,z directions decay with time, while the phase-plane trajectory remains confined within a bounded region and the Poincaré sampling points are concentrated within a small area. If the vibration amplitudes remain nearly unchanged and the phase-plane response shows no obvious expansion, the system is considered to be near the stability boundary. If the vibration amplitude in any direction continuously increases, accompanied by an expanding phase-plane trajectory and dispersed Poincaré sampling points, the system is regarded as unstable.

As shown in Figure 9 and Figure 11, and 13, under the operating conditions corresponding to Points A, C, and E, the vibration displacements of the cutter in the three directions exhibit relatively regular oscillatory behavior, with the vibration amplitudes gradually decreasing over time, indicating that the system response tends toward a stable state. In the corresponding x−y phase planes, the Poincaré sampling points are eventually concentrated within a small region, indicating that the cutter-tip displacement states remain similar when sampled at each tooth-passing period and that the system motion exhibits good periodic repeatability. Therefore, the milling processes at Points A, C, and E can be considered stable, which is consistent with their locations within the stable region of the stability lobe diagram.

As shown in Figure 10 and Figure 12, and 14, under the operating conditions corresponding to Points B, D, and F, the vibration displacement amplitudes in the three directions fluctuate significantly and exhibit an increasing trend over time, indicating that the system vibration cannot be effectively attenuated. In the corresponding x−y phase planes, the Poincaré sampling points are not concentrated within a confined region but instead show a relatively dispersed distribution. This indicates that the cutter tip cannot maintain similar displacement states over successive tooth-passing periods, and the periodic repeatability of the system motion is lost. Therefore, chatter occurs under these operating conditions, and the milling process is in an unstable state. This result is consistent with the prediction of the stability lobe diagram, in which Points B, D, and F are located in the unstable region.

### 3.4. Effect of Axial Contact Angle on Milling Stability

**Figure 15:**
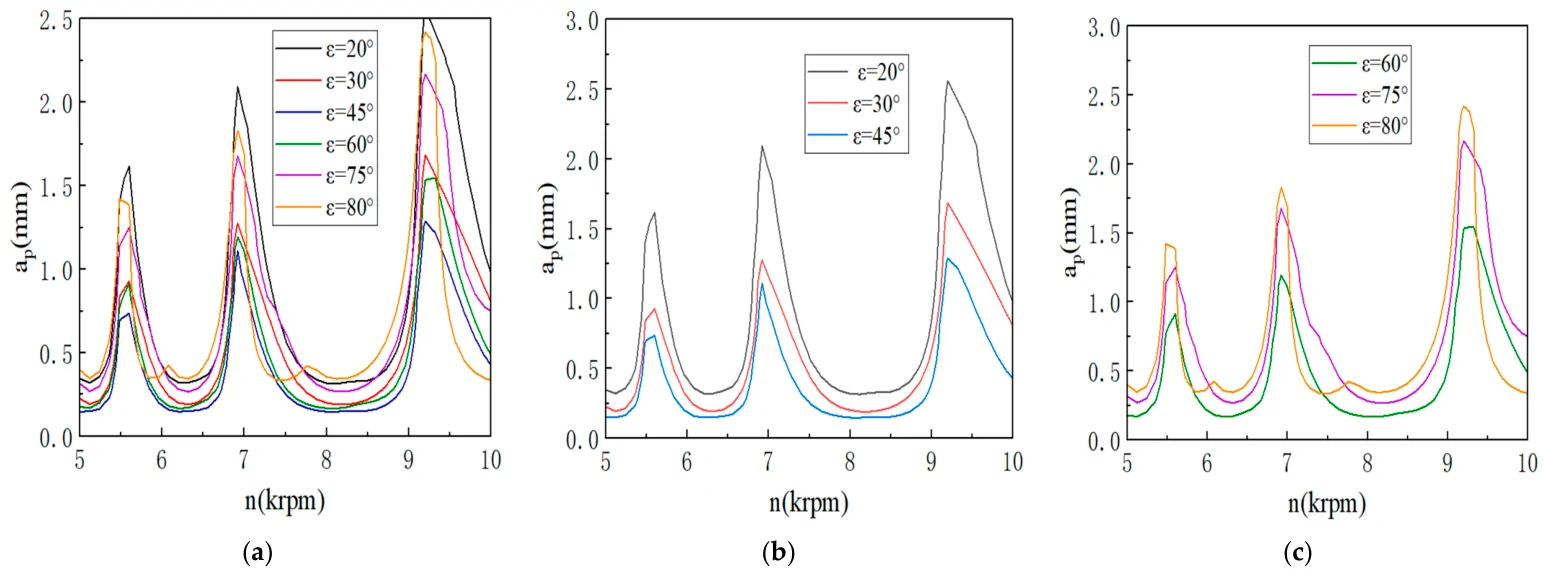
Stability lobe diagrams for different axial contact angles at m = 40 and a_e_/D = 1. (**a**) SLDs at different ε. (**b**) Region decreases with increasing ε. (**c**) Region increases with increasing ε.

**Figure 16:**
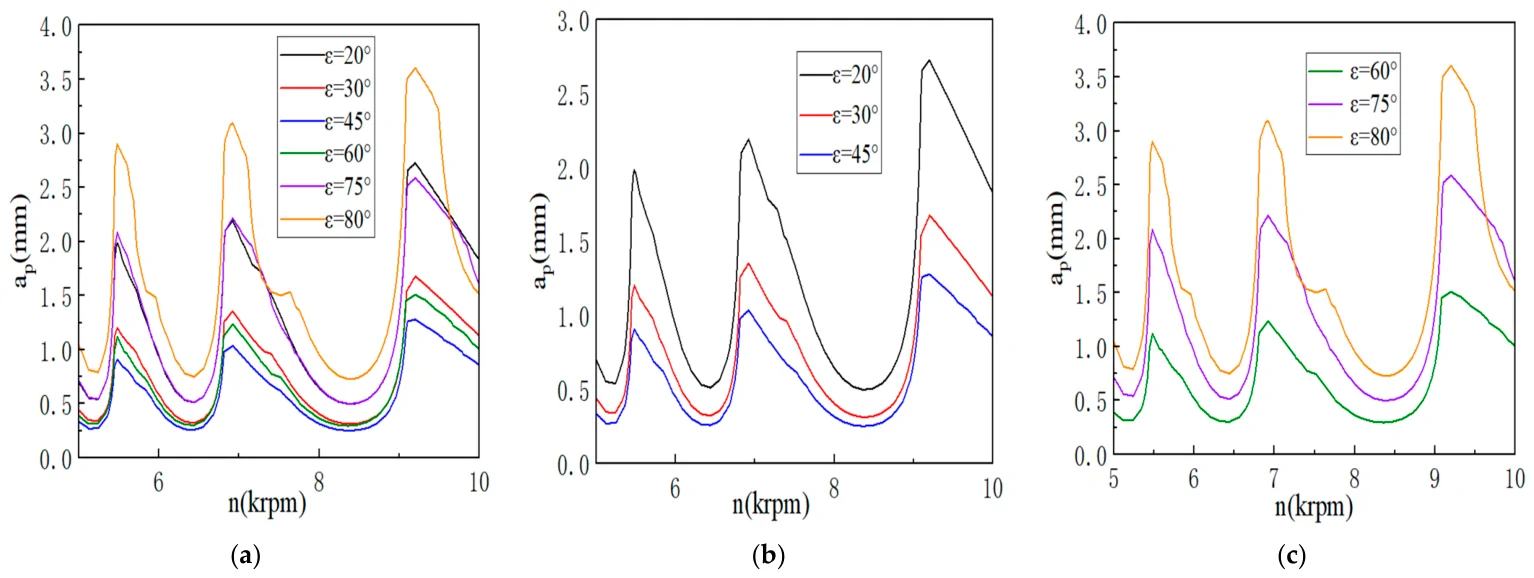
Stability lobe diagrams for different axial contact angles at m = 40 and a_e_/D = 0.5. (**a**) SLDs at different ε. (**b**) Region decreases with increasing ε. (**c**) Region increases with increasing ε.

**Figure 17:**
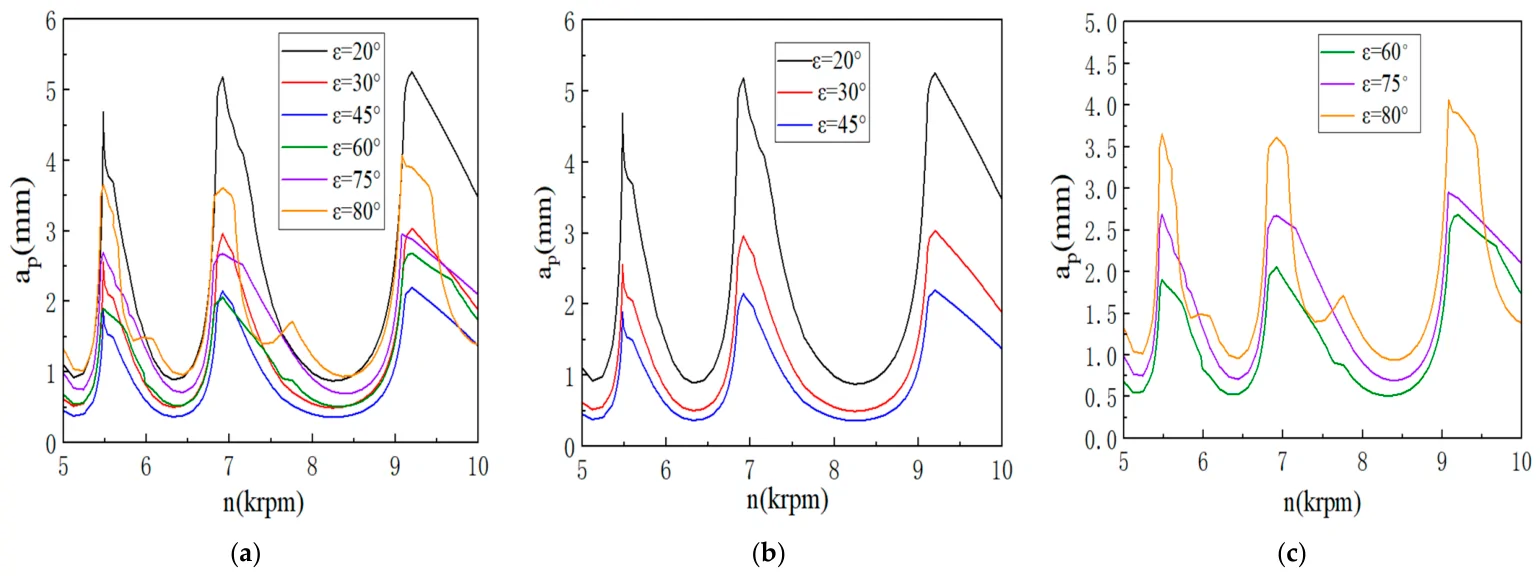
Stability lobe diagrams for different axial contact angles at m = 40 and a_e_/D = 0.1. (**a**) SLDs at different ε. (**b**) Region decreases with increasing ε. (**c**) Region increases with increasing ε.

During the milling process, different axial contact angles result in different dynamic characteristics of the milling system, which in turn lead to significant variations in the stability region. Figure 15, Figure 16 and Figure 17 show the stability regions obtained under different axial contact angles.

As shown in Figure 15, Figure 16 and Figure 17, the axial contact angle has a non-monotonic effect on the stability of the three-degree-of-freedom milling system under the conditions considered in this study. When the axial contact angle is relatively small, for example, $\varepsilon =20^{\circ }$, the axial contact geometric effect is relatively weak. The projection and coupling of cutting forces in the x, y, and z structural directions are limited, resulting in a weaker effective regenerative excitation and a relatively wider stability region.

As the axial contact angle increases, such as $\varepsilon =30^{\circ }$ and $\varepsilon =45^{\circ }$, the spatial projection relationships of the tangential, radial, and axial cutting forces in the three structural directions change accordingly, thereby modifying the coupling characteristics among the cutting-force components in different directions. Under the parameter conditions considered in this study, the enhanced directional coupling increases the effective regenerative excitation, leading to a gradual reduction in the stability region and a more pronounced tendency toward chatter.

When the axial contact angle further increases (e.g., $\varepsilon =60^{\circ }$, $\varepsilon =75^{\circ }$, and $\varepsilon =80^{\circ }$), the projection ratios and coupling relationships of the cutting forces in different structural directions undergo further changes. Since the contributions of cutting-force components in different directions to the system dynamic response are redistributed, their combined effect does not increase monotonically with the axial contact angle. Within the model and parameter range investigated in this study, the changes in force projection and coupling relationships may partially reduce the effective regenerative excitation, resulting in a certain recovery of system stability.

It should be noted that this non-monotonic trend is observed for the model and machining conditions considered in this study. The effect of the axial contact angle on milling stability may vary with the modal parameters, cutting-force coefficients, radial immersion, spindle speed, and milling mode. Therefore, both the extent of the stability-region variation and the corresponding turning points may change under different parameter combinations. The present results show that the axial contact angle affects the stability of the three-degree-of-freedom milling system mainly through changes in cutting-force transformation, force distribution, and coupling among the structural directions.

It can also be observed from Figure 15, Figure 16 and Figure 17 that the radial immersion ratio a_e_/D has a significant effect on cutting stability in the three-degree-of-freedom milling dynamic model. As a_e_/D increases, the dynamic excitation of the system is intensified, making chatter more likely to occur. Consequently, the stability region gradually shrinks, and the stable cutting boundary shifts toward smaller axial depths of cut and lower spindle speeds. The chatter-free cutting parameter range is continuously reduced, resulting in a marked decrease in the overall stability region.

## 4. Conclusions

Based on the third-order Lagrange–Hermite interpolation-based full-discretization method, the stability of a three-degree-of-freedom milling dynamic model considering axial geometric contact effects was analyzed and predicted. The main conclusions are summarized as follows.

(1) Under the consideration of axial contact angle and axial discretization, a three-degree-of-freedom milling dynamic model was established and formulated as a time-delay differential equation. The state term and delay term were discretized using third-order Lagrange interpolation and third-order Hermite interpolation, respectively. A state transition matrix was constructed, and system stability was determined based on Floquet theory.
(2) For the developed milling model, a comparative study on convergence performance was conducted between the proposed third-order Lagrange–Hermite interpolation-based full-discretization method, the first-order full-discretization method (1st-FDM), the second-order full-discretization method (2nd-FDM), and the third-order full-discretization method (3rd-FDM). The results indicate that the proposed method achieves faster convergence and higher numerical accuracy under different axial depths of cut.
(3) The effects of axial discretization and axial contact angle on the predicted stability lobe diagrams (SLDs) were investigated separately. The results show that, within the parameter ranges of spindle speed n = 5000–10,000 rpm and axial depth of cut a_p_ = 0–8 mm, the overall variation rate of the predicted stability region is 11.19% when axial discretization is considered and 59.88% when the axial contact angle is incorporated. In addition, the Stable Area Ratio (SAR) was evaluated under different radial immersion ratios and milling strategies, further demonstrating the necessity of considering axial geometric contact effects in milling stability prediction.
(4) Time-domain simulations were conducted to obtain the three-directional displacement responses and phase-plane trajectories of the cutter under representative cutting conditions, thereby validating the effectiveness of both the proposed third-order Lagrange–Hermite interpolation-based full-discretization method and the milling model incorporating axial geometric contact effects. Further analysis of the stability regions under different axial contact angles reveals that the axial contact angle has a significant nonlinear influence on milling stability.

The above results demonstrate the numerical accuracy, convergence performance, and consistency of the proposed stability prediction framework. The developed model further quantifies the effects of axial contact angle variation and axial discretization on the predicted stability regions. It should be noted that the predicted stability boundaries and their modifications for the complete three-degree-of-freedom milling model have not yet been independently validated through milling experiments, which represents a limitation of the present study. Therefore, the reported changes in the stability regions should primarily be interpreted as quantitative differences between different modeling conditions, while their physical accuracy requires further experimental assessment. Within this scope, the proposed approach provides a theoretical and numerical basis for milling stability analysis and the selection of appropriate cutting parameters.

## Figures and Tables

**Table 1:** Tool geometry parameters of the three-degree-of-freedom milling system.

D(mm)	$N_{t}$	ε	n(krpm)	$a_{p}(\mathrm{mm})$	$a_{e}(\mathrm{mm})$	$f_{z}(\mathrm{mm}/\mathrm{tooth})$
10	2	75°	5–10	0–8	1–10	0.5

**Table 2:** Simulation parameters of the three-degree-of-freedom milling system.

$f_{n}\left(\mathrm{Hz}\right)$	ζ	$m_{t}(\mathrm{kg})$	$K_{\mathrm{tc}}\left(N/m^{2}\right)$	$K_{\mathrm{rc}}\left(N/m^{2}\right)$	$K_{\mathrm{ac}}\left(N/m^{2}\right)$	$K_{\mathrm{te}}\left(N/mm\right)$	$K_{re}\left(N/mm\right)$	$K_{ae}\left(N/mm\right)$
922	0.011	0.03993	6 × 10^8^	2 × 10^8^	2 × 10^8^	100	70	40

## Data Availability

The original contributions presented in the study are included in the article; further inquiries can be directed to the corresponding author.

## Nomenclature

$f_{t}$	Feed per tooth
T	time delay
$N_{t}$	Number of teeth
n	Spindle speed
$\phi_{\mathrm{st}}$	Entry angle of the cutter tooth
$\phi_{ex}$	Exit angle of the cutter tooth
$a_{e}/D$	Ratio of radial depth of cut to cutter diameter
$a_{p}$	Axial depth of cut
M	Modal mass matrix of the system
C	Modal damping matrix of the system
K	Modal stiffness matrix of the system
$A_{0}$	Constant system matrix
$A\left(t\right)$	Time-varying current-state matrix
$B\left(t\right)$	Time-varying delayed-state matrix
I	Identity matrix
τ	Discretization time step
k	Discrete-step index
m	Number of discrete intervals
D	Cutter diameter
ε	Axial contact angle
$a_{e}$	Radial depth of cut
$f_{n}$	Natural frequency
ζ	Damping ratio
$m_{t}$	Modal mass of the cutting tool
$K_{\mathrm{tc}}$	Tangential cutting-force coefficient
$K_{\mathrm{rc}}$	Radial cutting-force coefficient
$K_{\mathrm{ac}}$	Axial cutting-force coefficient
$K_{\mathrm{te}}$	Tangential edge-force coefficient
$K_{\mathrm{re}}$	Radial edge-force coefficient
$K_{\mathrm{ae}}$	Axial edge-force coefficient
$\left|\mu \right|$	Approximate spectral radius
$\left|\mu_{0}\right|$	Reference spectral radius
DDE	Delay differential equation
SLD	Stability Lobe Diagram
1st-FDM	First-order full-discretization method
2nd-FDM	Second-order full-discretization method
3rd-FDM	Third-order full-discretization method
3rd-LH-FDM	Third-order Lagrange–Hermite interpolation-based full-discretization method
